# Colchicine attenuates the electrical remodeling of post-operative atrial fibrillation through inhibited expression of immune-related hub genes and stabilization of microtubules

**DOI:** 10.7150/ijbs.81961

**Published:** 2023-06-04

**Authors:** Hangying Ying, Wenpu Guo, Xiaomei Tang, Jun Pan, Pengcheng Yu, Hangping Fan, Xiaochen Wang, Ruhong Jiang, Chenyang Jiang, Ping Liang

**Affiliations:** 1 Department of Cardiology, Sir Run Run Shaw Hospital, Zhejiang University School of Medicine, Hangzhou 310016, China.; 2 Key Laboratory of Cardiovascular Intervention and Regenerative Medicine of Zhejiang Province, Hangzhou 310016, China.; 3 Department of Breast Surgery, Second Affiliated Hospital, Zhejiang University School of Medicine, Hangzhou 310009, China.; 4 Key Laboratory of Tumor Microenvironment and Immune Therapy of Zhejiang Province, Hangzhou 310009, China.; 5 Key Laboratory of combined Multi-organ Transplantation, Ministry of Public Health, First Affiliated Hospital, Zhejiang University School of Medicine, Hangzhou 310003, China.; 6 Institute of Translational Medicine, Zhejiang University, Hangzhou 310029, China.

**Keywords:** colchicine, post-operative atrial fibrillation, epicardial adipose tissue, bioinformatics, hub genes

## Abstract

**Rationale:** Acute inflammation is a major risk factor for post-operative atrial fibrillation (POAF), and epicardial adipose tissue (EAT) is considered as a source of inflammatory mediators. However, underlying mechanisms and pharmacological targets of POAF are poorly understood.

**Methods:** Integrative analysis of array data from EAT and right atrial appendage (RAA) samples was conducted to identify potential hub genes. Lipopolysaccharide (LPS)-stimulated inflammatory models in mice and in induced pluripotent stem cell-derived atrial cardiomyocytes (iPSC-aCMs) were used to examine the exact mechanism underlying POAF. Electrophysiological analysis, multi-electrode array, and Ca^2+^ imaging was employed to explore the alterations of electrophysiology and Ca^2+^ homeostasis under inflammation. Flow cytometry analysis, histology and immunochemistry were performed to investigate immunological alterations.

**Results:** We observed electrical remodeling, enhanced atrial fibrillation (AF) susceptibility, immune cell activation, inflammatory infiltration, and fibrosis in LPS-stimulated mice. LPS-stimulated iPSC-aCMs showed arrhythmias, abnormal Ca^2+^ signaling, reduced cell viability, disrupted microtubule network and increased α-tubulin degradation.* VEGFA*, *EGFR*, *MMP9* and *CCL2* were identified as hub genes simultaneously targeted in the EAT and RAA of POAF patients. Notably, treatment of colchicine in LPS-stimulated mice resulted in a U-shape dose-response curve, where greatly improved survival rates were observed only at doses between 0.10-0.40 mg/kg. At this therapeutic dose level, colchicine inhibited the expression of all the identified hub genes and effectively rescued the pathogenic phenotypes observed in LPS-stimulated mice and iPSC-aCM models.

**Conclusions:** Acute inflammation promotes α-tubulin degradation, induces electrical remodeling, and both recruits and facilitates the infiltration of circulating myeloid cells. A certain dose of colchicine attenuates electrical remodeling and decreases the recurrence of AF.

## Introduction

Post-operative atrial fibrillation (POAF), the most common perioperative cardiac arrhythmia, affects 20-40% patients with cardiac surgical procedures and 10-20% with non-cardiac thoracic operations^1^. POAF is generally a transient phenomenon with peaking incidence at 2-4 days after surgery. It results in lengthened hospital and intensive care unit stays and incurs additional treatment costs^1^-^3^. As the most important secondary atrial fibrillation (AF), the pathogenic mechanisms of POAF include electrical remodeling, structural remodeling, Ca^2+^ handling abnormalities and autonomic nervous system changes^4^. Due to incomplete understanding of the pathogenesis of POAF, many of the proposed preventive approaches lack sufficient efficacy and are further complicated by complex interplay between the pre-existing physiological components and local and systemic inflammation may involve in the etiology of POAF^2^,^3^,^5^,^6^. Acute inflammation is a major transient post-operative factor that may trigger POAF^2^,^5^, particularly where excessive inflammation increases the atrial conduction heterogeneity^7^. The activation and infiltration of specific immune cells and the presence of inflammation markers could therefore be used to predict AF onset in patients having undergone operative surgery^8^.

Epicardial adipose tissue (EAT) is the fat depot situated between the atrioventricular and interventricular grooves and along the coronary arteries. As a metabolically active tissue, EAT produces pro-inflammatory and pro-fibrotic adipokines^9^,^10^, and as such has been long considered as a source for the inflammatory mediators of cardiovascular disease^9^. Growing evidence increasingly suggests the role EAT induced inflammation in the pathophysiology of POAF and secreted proteins and adipokines from EAT have now been characterized as a potential substrate in the development of POAF^11^-^13^. Pharmacotherapy that exerts anti-inflammatory roles that simultaneously target both EAT and atrial tissues would therefore be a valid therapeutic strategy for POAF^7^,^14^,^15^. Colchicine is a natural and sophisticated anti-inflammatory agent that has been used for decades as a staple therapy for gout and as a second-line treatment for pericarditis^16^. Colchicine has unique anti-inflammatory properties that differ from those of non-steroidal anti-inflammatory drugs and glucocorticoids, particularly as it does not affect the arachidonic acid pathway but instead acts directly on microtubules^17^.

However, the two best-known consecutive studies assessing colchicine for the prevention of POAF had failed to provide effective and consistent results due to premature study discontinuation resulted from adverse events (COPPS-1 and COPPS-2)^18^,^19^. According to the American College of Cardiology (ACC)/American Heart Association (AHA) guidelines, the use of colchicine for preventing POAF has an evidence class of IIB^20^. It is therefore crucial to determine whether colchicine could be effective in preventing POAF and how it could be used for better clinical outcomes.

In the present study, we aimed to investigate the electrophysiological and immunological alterations observed during the progress of acute inflammation in POAF. We also aimed to identify therapeutic targets and effective agents relevant to POAF.

## Methods

### Data availability and processing

Raw array data were obtained for data analysis from the Gene Expression Omnibus database (GEO submission: GSE143924, GSE62871) **Supplemental Table 1**. The raw data of GSE143924 were preprocessed using the robust multi-array average (RMA) algorithm with the 'affy' package. This process included background correcting, normalizing, and calculating expression. For the raw data of GSE62871, the background was subtracted using the function backgroundCorrect, and data normalization was conducted using the functions normalizeWithinArrays and normalizeBetweenArrays.

### Analysis of immune cluster characterization and cell abundance

Bioinformatics algorithms (CIBERSORT, ESTIMATE) were used to evaluate the immune cluster characterization and cell abundance between POAF and sinus rhythm (SR) patients from EAT and the right atrial appendage (RAA). We used CIBERSORT, a deconvolution algorithm, to characterize different immune cell compositions of EAT and the RAA from their gene expression profiles^21^. The ESTIMATE algorithm was applied to calculate the proportion of immune and stromal components in the immune microenvironment where the immune and stromal scores reflect inflammatory cell infiltration^22^.

### Weighted correlation network analysis

Weighted gene co-expression network analysis (WGCNA) was used to group highly correlated genes and to attach to external sample traits^23^. The top 25% of the variances in the GSE143924 (EAT) and GSE62871 (RAA) were selected for respective immune cluster characterization. The 'WGCNA' package was used to select an appropriate soft-thresholding power β to achieve scale-free topology^23^. Selected genes were clustered into exclusive co-expressing modules, each module depicted with different built-in colors using the average linkage hierarchical clustering method. To improve the reliability of the results, the minimum number of genes in each module was 100 and the threshold for module merging was 0.25 in GSE62871. In the WGCNA package, we used the Pearson's correlation method to calculate the correlation between each exclusive module and the immune cell abundance as identified by CIBERSORT.

### Functional enrichment analysis

Metascape (http://metascape.org/) is a web-based portal designed to enable integrative, end-to-end analysis of OMICs-level datasets. This tool facilitates comparisons of datasets from multiple independent and orthogonal experiments^24^. Metascape was performed to analyze functional profiles of modules most associated with POAF. An adjusted *p*< 0.05 was considered statistically significant.

### Construction of protein-protein interaction network and identification of hub genes

The pathogenic targets of POAF were searched using Online Mendelian Inheritance in Man (OMIM, http:// omim.org/)^25^ and Gene Cards (http://www.genecards.org/) databases^26^. The intersection of MEturquoise, POAF-related genes, and all genes detected in GSE62871, was imported into the Search Tool for the Retrieval of Interacting Genes (STRING, v11.0) to generate a protein-protein interaction (PPI) network^27^. Results were visualized using Cytoscape software (v3.8.2). The plug-in cytoHubba was applied to identify hub genes based on degree^28^. The intersections of the hub genes obtained by CytoHubba were considered as potential anti-inflammatory drug targets.

### Establishment of inflammation mouse model and treatment protocol

All animal studies were conducted with approval from the Animal Welfare Ethics Committee of Sir Run Run Shaw Hospital, Zhejiang University School of Medicine. The specific experimental steps of mice are shown in Figure 4A. Male C57BL/6 mice aged 8 weeks were injected intraperitoneally with 15.0 mg/kg lipopolysaccharide (LPS, Escherichia coli 0111: B4, L2630, Sigma-Aldrich) dissolved in normal saline (NS). Mice were subjected to an equal volume of NS as controls. For the LPS + colchicine group, colchicine was solubilized in dimethyl sulfoxide (DMSO) and diluted with NS to different concentrations. Colchicine was administered by gavage 12 hours before LPS injection. For LPS + AG-1478 group, AG-1478 was solubilized in DMSO and diluted with NS to a final DMSO concentration of < 0.1%. AG-1478 was then intragastrically administrated once a day for 10 consecutive days before LPS injection. Survival time was monitored continuously for a total of 72-hour period after LPS injection. Mice were anesthetized with 60 mg/kg of 1% sodium pentobarbital before sacrifice. Whole hearts were then rapidly excised, and atria subsequently removed from each heart. Atria tissues were stored at -80℃ for further analysis.

### Electrophysiological analysis

Electrocardiogram (ECG) data was obtained from mice as performed using surface electrocardiogram and programmed intracardiac stimulation^29^. Mice were anesthetized with 60 mg/kg of 1% sodium pentobarbital. Electrode needles were inserted subcutaneously into the left, right upper limb and right lower limb for ECG recording (iWorx system, IX-RA-B3G). The ECG waveforms were monitored until a stable baseline and heart rate were reached. Every baseline ECG was recorded for 3 minutes. Programmed intracardiac stimulation was then applied to assess AF inducibility^29^,^30^. Briefly, a 1.1 French 8-polar electrodes catheter (Electrophysiology Control Unit & Catheters, Transonic science) was advanced through the vein into the right atrium. The exact catheter position was confirmed by visualization of the waveforms of intracardiac electrograms. AF was then induced by an overdrive pacing protocol with decremental burst pacing using an automated stimulator. The protocol began with 2-second burst pacing at a cycle length of 40 ms, then decreasing in each successive burst by a 2-ms decrement to a cycle length of 20 ms^30^.

Successful AF was defined as the occurrence of rapid and fragmented atrial electrograms with irregular R‐R intervals lasting for at least 2 seconds. To ensure the reliability of the results, AF induction protocols were typically performed in triplicate, and an AF was considered present if it was recorded in at least two out of three trials. The total duration time of each AF episode was defined as the sum of each AF episode time. ECG traces were analyzed using the LabScribe 4.361 (iWorx system).

### Plasma Isolation and enzyme linked immunosorbent assay

Mouse blood was collected in 1.0 mL quantities using an EDTA tube and kept at 4°C for 24 hours. Plasma was isolated through centrifugation at 3000 rpm for 15 minutes and stored at -80°C until use. For *in vivo* studies, the concentrations of mouse plasma of tumor necrosis factor α (TNF-α, SEA133Mu 48T, USCN life), and interleukin 6 (IL6, SEA079Mu 48T, USCN life) were determined using enzyme linked immunosorbent assay (ELISA) kits according to the manufacturers' instructions. For *in vitro* studies, the intracellular IL6 contents were measured using an ELISA kit for IL6 (IL6, SEA079Hu 48T, USCN life).

### Flow Cytometry Analysis

To analyze the immune cell components in the blood and atrial tissues between differently treated groups, peripheral blood and atrial tissues in differently treated mice were obtained. Single cell suspensions from peripheral blood (PB) and atrial tissues were made as previously reported^31^. The cells were stained with Zombie Red Fixable Viability Kit (BioLegend) or 7-AAD (BioLegend) to exclude dead cells. Live cells were then stained with fluorescein-conjugated monoclonal antibodies and analyzed by FACS. For lymphoid cells sorting, the antibodies were CD45-APC/Cy7, CD3-PE/Cy7, CD20-BV421 (BioLegend). For myeloid cell sorting, the antibodies were CD45-BV510, CD11b-PE/Cy, CD11c-AF700, Ly-6G-Percp/Cy5.5, Ly-6G-Percp/FITC, F4/80 ECD (BioLegend), and were analyzed using a BD FACS Fortessa. All flow cytometry data were analyzed using FlowJo software (FlowJo V10, LLC).

### Histology and immunochemistry analysis

At the end of the experiment, mice were sacrificed under deep anesthesia followed by the rapid excision of the heart and peripheral blood. The atria were removed from each heart and fixed in 4% paraformaldehyde (PFA) (Beyotime). Paraffin-embedded atrial sections were stained with hematoxylin-eosin (HE) to evaluate the degree of inflammatory infiltration and myocardial damage under intense inflammation. Sections of atria also were stained with Masson's trichrome to evaluate the distribution and localization of collagen.

Immunohistochemistry (IHC) analysis was performed on paraffin-embedded sections of atria tissues. Paraffin-sections were sequentially subjected to dewax, rehydrate, antigen-repaire, block endogenous peroxidase activity, and serum seal. The primary antibodies used were EGFR antibody (Servicebio, 1:1000), CD3 antibody (Servicebio, 1:300), CD20 antibody (Servicebio, 1:300), F4/80 antibody (Servicebio, 1:700) and Tryptase (Abcam, 1:100) which were added and incubated with sections overnight at 4℃. The corresponding species of primary antibody were added and incubated at room temperature for 50 minutes after three time washing with PBS. After DAB chromogenic reaction, nucleus counterstaining and mounting, images were then obtained using an automatic digital slide scanning system (KFBIO, KF-PRO-120) and analyzed using ImageJ.

### Culture and maintenance of iPSCs

Induced pluripotent stem cells (iPSCs) were cultured and maintained as previously described^32^,^33^. Briefly, iPSCs were cultured in feeder-free mTeSR1 (STEMCELL Technologies) media on matrigel (Corning)-coated plates at 37°C with 5% (vol/vol) CO_2_. The media were daily changed, and iPSCs were passaged every 3-4 days using Accutase (STEMCELL Technologies).

### Alkaline phosphatase staining

Alkaline phosphatase (ALP) staining was performed using a VECTOR Blue Alkaline Phosphatase Substrate Kit (Vector Laboratories) following the manufacturer's instructions.

### Differentiation of iPSC-derived atrial cardiomyocytes

The iPSCs were differentiated into cardiomyocytes using a 2D monolayer differentiation protocol. Briefly, ~10^5^ undifferentiated cells were dissociated and re-plated into matrigel-coated 6-well plates. Cells were cultured and expanded to 85% cell confluence and then treated for 2 days with 6 μM CHIR99021 (Axon Medchem) in RPMI and B-27 supplement minus insulin (RPMI+B27-Insulin) (Gibco) to activate the Wnt signaling pathway. On day 2, cells were placed in RPMI+B27-Insulin with CHIR99021 removal. On days 3-4, cells were treated with 5 μM IWR-1 (Merck) to inhibit the Wnt signaling pathway. Stocks of retinoic acid (RA) were prepared as 2 mM in DMSO. On day 5-6, differentiated cells were removed from the IWR-1 treatment and placed in RPMI+B27-Insulin+2 μM RA to atrial myocytes. From day 7 onwards, cells were placed and cultured in RPMI and B-27 supplement with insulin (RPMI+B27+Insulin) (Gibco) until beating was observed. Cells were glucose-starved for 3 days with RPMI+B27+Insulin for purification. The iPSC-derived atrial cardiomyocytes (iPSC-aCMs) of day 30-40 after cardiac differentiation were utilized for downstream investigations.

### Identification of human iPSC-aCMs

The iPSC-aCMs were identified via their action potential morphology and action potential parameters using patch clamp. The iPSC-aCMs were mechanically and enzymatically dissociated to obtain single cells, which were then seeded on Matrigel-coated glass coverslips (Warner Instruments). Cells with spontaneous beatings were selected and action potentials were recorded using an EPC-10 patch clamp amplifier (HEKA). Continuous extra-cellular solution perfusion was achieved using a rapid solution ex-changer (Bio-logic Science Instruments). Data were acquired using the PatchMaster software (HEKA) and digitized at 1kHz. Data analyses were performed using Igor Pro (Wavemetrics) and Prism-9 (GraphPad Software). A TC-344B heating system (Warner Instruments) was used to maintain the temperature at 35.5-37 °C. The external solution contained 140 mM NaCl, 5.4 mM KCl, 1 mM MgCl_2_, 10 mM glucose, 1.8 mM CaCl_2_ and 10 mM HEPES (pH 7.4 with NaOH at 25 °C). The internal solution contained 120 mM KCl, 1 mM MgCl_2_, 10 mM HEPES, 3 mM Mg-ATP, and 10 mM EGTA (pH 7.2 with KOH at 25 °C).

### Cell viability assay

The iPSC-aCMs were plated into a 96-well plate with 4.5×10^5^ cells per well. After drug treatment, cell viability assays were performed using Cell Counting Kit-8 (CCK-8) kits (Beyotime) according to the manufacturer's instructions. Absorbance was measured using a microplate reader (BioRad iMark^TM^ Microplate Reader) at 450 nm.

### Multi-electrode array (MEA)

Experiments and analyses were conducted as previously described^32^. Briefly, 1-well or 6-well MEA probes (60MEA 200/30iR-Ti-gr or 60-6wellMEA200/30iR-Ti-tcr, Multi Channels Systems) were preprocessed using coating solution (matrigel). The iPSC-aCMs were seeded in the central recording wells at a density of 2.0-2.5×10^5^ cells for each probe. The medium was changed every 2 days until the beginning of recording. Field potentials were recorded from spontaneously beating iPSC-aCMs using the MEA2100 data acquisition system (Multi Channel Systems) with sampling at 10 kHz. All experiments were conducted at 37°C and began after a 10-minute equilibration period. The measured beating period (BP), field potential duration (FPD), and field potential amplitude (FPA) were analyzed using Cardio 2D^+^ software (Multi Channel Systems). As the FPD is inversely related to beating rate, the FPD was adjusted for the beating rate to yield corrected FPDc. Steady-state parameters were then averaged and the FPD was normalized to the beat rate using the Fridericia's correction formula: FPDc= FPD/(inter-spike interval)^1/3^, where inter-spike interval (ISI) indicates the time interval (in seconds).

### Ca^2+^ imaging

The iPSC-aCMs grown on coverslips were loaded with RPMI 1640 medium without phenol Red (Invitrogen) supplemented with 5 μM Fura-2 AM (the stock of Fura-2 AM was pre-dissolved in 20% Pluronic F-127 solution in DMSO) for 30 minutes in the dark at room temperature. After twice washing with pre-warmed DPBS and RPMI 1640, the cells were immersed in imaging buffer for 30 minutes before experiments. Fluorescent signals were obtained upon excitation at 340 nm (F_340_) and 380 nm (F_380_). Amplitude of Ca^2+^ transient is defined as the ratio of F_340_/F_380_.

### Immunoblotting analysis

Cells and tissues were lysed using radioimmunoprecipitation assay (RIPA) lysis buffer (Solarbio) with a protease-inhibitor cocktail (Roche) and a phosphatase- inhibitor cocktail (Roche). Total protein concentrations were normalized by BCA without boiling. Western blot was performed using standard protocol with the following primary antibodies: RyR2 (Abcam, ab2868, 1:1000), RyR2-phospho Ser2814 (Badrilla, A010-31AP, 1:500), CaMKII (Abcam, ab181052, 1:1000), CaMKII-phospho T286 (Abcam, ab32678, 1:1000), Ca_v_1.2 (Abcam, ab84814, 1:1000), NCX1 (Proteintech, 55075-1-AP, 1:1000), SERCA2a (Santa Cruz Biotechnology, sc-53010, 1:200), and GAPDH (Abmart, M200006, 1:5000).

### Quantitative real-time PCR (qPCR)

RNA was isolated using TRIzol (Ambion) according to the instruction manual. The RNA concentration was measured using UV spectrophotometry at 260 nm (Nanodrop 2000, Thermo Scientific). Complementary DNA (cDNA) was obtained from mRNA using a PrimeScript RT reagent kit (Takara). qPCR was performed using SYBR Green PCR Master Mix (EZBioscience). The primer sequences used in this study are listed in **Supplemental Table 2**. Each reaction was run in triplicate using an Applied Biosystems QuantStudio 6 Flex (Thermo Fisher Scientific). Gene expression values were normalized to the average expression of the housekeeping gene *GAPDH*.

### Immunofluorescence staining

Cells were fixed with 4% PFA for 10 minutes, permeabilized with 0.1% Triton X (Sangon Biotech) for 10 minutes at room temperature and blocked with 3% BSA (Sigma-Aldrich) for 1 hour. Cells were then washed and subsequently stained with the appropriate primary antibodies and AlexaFluor conjugated secondary antibodies (Life Technologies). Nuclei were stained with DAPI (Roche Diagnostics) or Hoechst (Solarbio, HOE 33258). The primary antibodies of OCT4 (Santa Cruz Biotechnology), NANOG (Santa Cruz Biotechnology), SSEA-4 (Abcam), and SOX2 (Abcam) were stained as pluripotency markers. The primary antibodies of TNNT2 (Abcam) and α-actinin (Abcam) were stained as cardiac-specific markers. The NR2F2 (Abcam) and MLC2a (Abcam) were staining as atrial-specific markers. The α-tubulin in microtubule network was stained with Tubulin-Tracker^TM^ Deep Red (Sigma-Aldrich, T34077) and β-tubulin was stained with Tubulin-Tracker green staining kit (Beyotime, C22135). Pictures were obtained with 60× objective on confocal microscope (Nikon, A1) using NIS-Elements AR software (Nikon).

### Statistical Analysis

Results are presented as mean ± standard deviation (SD). Normality was evaluated using D'Agostino & Pearson omnibus testing. The F-test in Prism-9 (GraphPad Software) was used to verify the homogeneity of variance. Quantitative data were analyzed using unpaired t-tests, or the Mann-Whitney tests if the normality test failed. The incidences of AF (%) and arrhythmias (%) was analyzed using the Fisher' exact probability test. Survival analysis was performed using Kaplan-Meier survival curves. A value of *p*< 0.05 was considered statistically significant. ^*^p< 0.05, ^**^p< 0.01, ^***^p< 0.001 and ^****^p< 0.0001.

## Results

### Comparison of immune cluster characterization and cell abundance between EAT and the RAA

**Figure 1:**
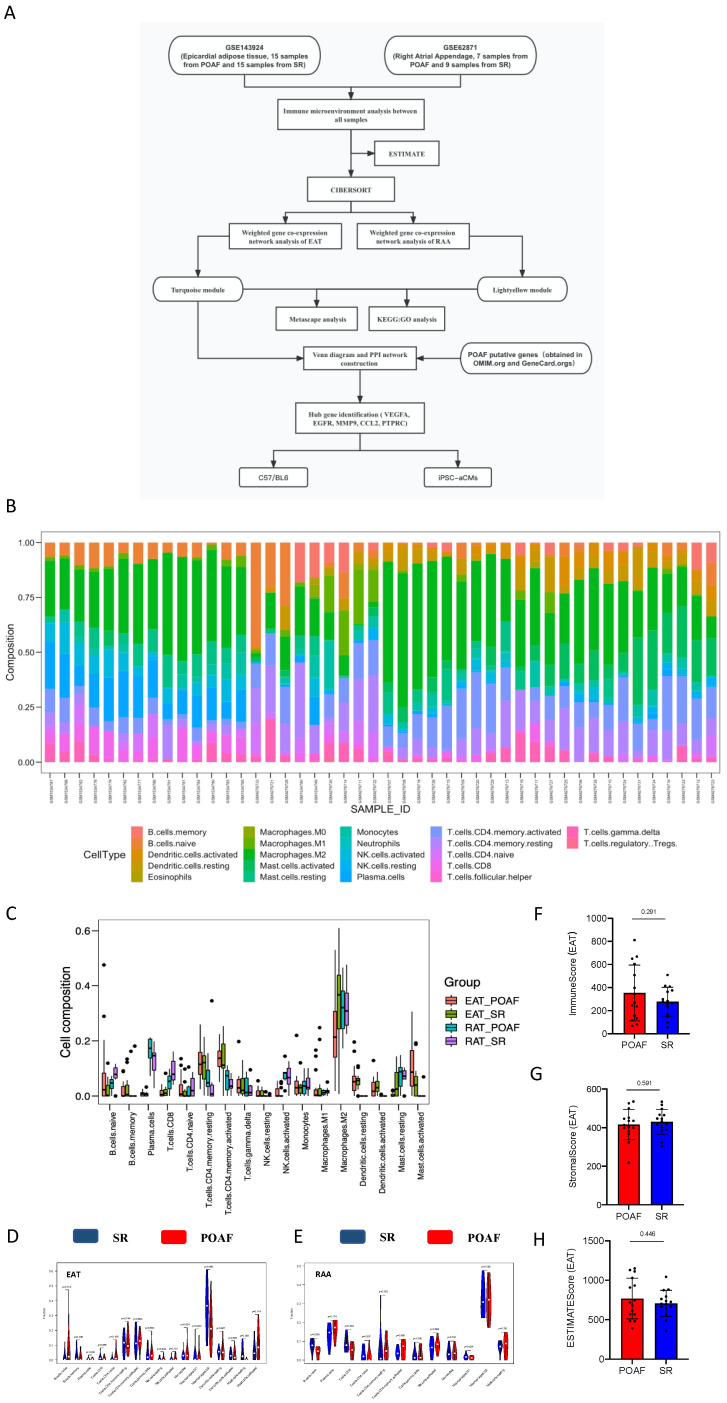
** Comparison of immune cluster characterization and cell abundance between EAT and the RAA. A.** Flowchart of bioinformatics analysis. **B.** Relative expression of 22 immune cell abundance stacked column charts in EAT and RAA samples estimated using CIBERSORT. **C-E.** Immune cluster characterization and cell abundance histogram of all included samples, with EAT samples and RAA samples using CIBERSORT. **F-H.** Immune, stromal, and estimate scores of EAT samples using ESTIMATE.

To explore the aberrant alterations of immune status between POAF and SR patients we performed bioinformatics algorithms to evaluate the immune cluster characterization and cell abundance in EAT and RAA samples (**Figure 1A**). Detailed information of all included data is provided in **Supplemental Table 1**, and bar graphs representing before and after quality control are presented in **Supplemental Figure 1A-D**. According to the CIBERSORT algorithm, T cells, M2 macrophages and mast cells represent the majority categories among all types of immune abundances; with B cells, NK cells and eosinophils representing only a small minority **(Figure 1B)**. Although no statistical significance was observed between multiple immune cells due to inter-individual variability, our results suggest that T lymphocytes, B lymphocytes, macrophages, and mast cells may contribute to the recurrence of POAF **(Figure 1B)**. As shown in **Figure 1C-E**, the different subtypes of T cells varied not only between POAF and SR patients, but also in EAT and the RAA. The contents of Naïve B cells in POAF patients showed an increased trend in EAT** (***p*= 0.174, **Figure 1D)**, but were significantly decreased in the RAA **(***p*= 0.039, **Figure 1E)**. Notably, M2 macrophages accounted for the highest proportion of all immune cells simultaneously targeted in EAT and the RAA, for both SR and POAF patients in combination **(Figure 1D-E)** and was only shown to be specifically decreased in the EAT of POAF patients **(***p*= 0.05,** Figure 1D)**. Mast cells have been considered as pro-fibrotic mediators in the development of AF^34^. We observed that in EAT, the proportion of activated mast cells had increased (*p*= 0.114) and the proportion of resting mast cells decreased (*p*= 0.083) in POAF patients, when compared to SR patients **(Figure 1D)**. However, the proportions of mast cells in the RAA were comparable between POAF and SR patients **(***p*= 0.791, **Figure 1E)**. Similar to the biological properties of tumors, EAT gradually infiltrates the contacted myocardium, releasing proinflammatory and profibrotic cytokines and chemokines as it does so^35^. ESTIMATE results demonstrated higher immune scores in POAF patients than those in SR patients **(***p*= 0.291, **Figure 1F)**, while no differences were observed for stromal score **(***p*= 0.591, **Figure 1G)** or estimate score **(***p*= 0.446, **Figure 1H)**.

### Construction of the weighted co-expression network and identification of the key modules

**Figure 2:**
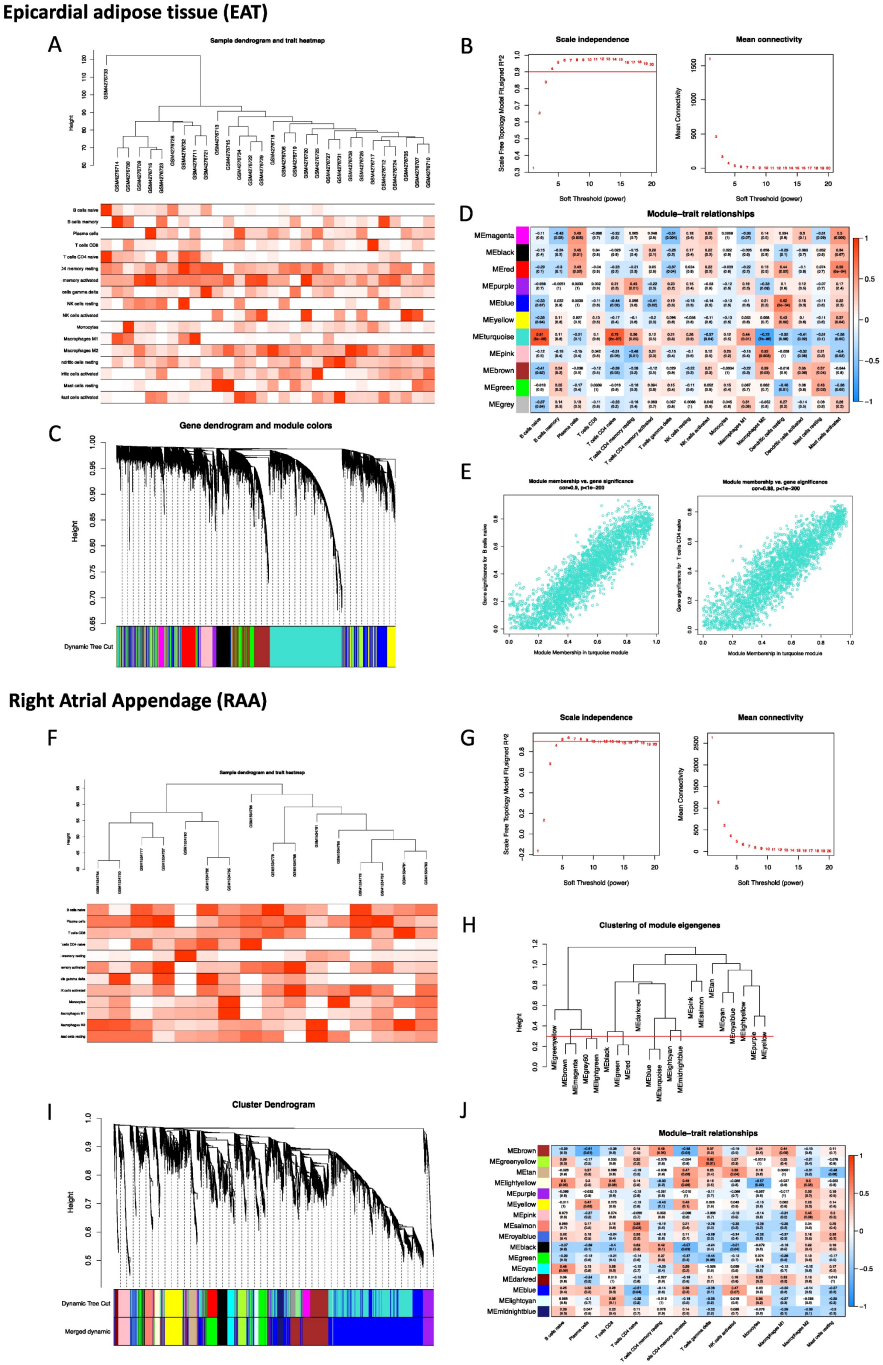
** Construction of the weighted co-expression network and identification of key modules. A.** Sample dendrogram and trait heatmap of EAT. **B.** Selection of the soft-thresholding power β in EAT analysis. **C.** Dendrogram of all selected genes in EAT. **D.** Heatmap of module-trait relationships for EAT in the constructed network. **E.** Scatter diagrams for module membership vs. gene significance of the disease state in the turquoise module. **F.** Sample dendrogram and trait heatmap of the RAA. **G.** Selection of the soft-thresholding power β in RAA analysis. **H.** Clustering diagram of module eigengenes. **I.** Dendrogram of all selected genes in the RAA. **J.** Heatmap of module-trait relationships of the RAA in the constructed network.

To robustly group correlated genes closely related to the presence of POAF, WGCNA was performed twice using two different datasets, GSE143924 (EAT) and GSE62871 (RAA). In analysis of EAT, the cut-height was 40, and one sample (GSM4276733) was excluded as an outlier **(Figure 2A)**. The soft-threshold β= 4 was applied to construct a gene co-expression network **(Figure 2B)**, with the majority of the genes in the turquoise module **(Figure 2C)**. Given the fact that Naïve B cells, CD4 Naïve T cells, M2 macrophages and mast cells had been identified as the most relevant immune cells corresponding to the incidence of POAF, the turquoise module was therefore selected for subsequent experiments **(Figure 2D)**. The representative correlation coefficient values in the turquoise module are visualized in **Figure 2E**. In the analysis of the RAA, the cut-height was 65 and no sample was excluded from subsequent analysis **(Figure 2F)**. The soft threshold β= 5 was applied to construct a gene co-expression network **(Figure 2G)**. 0.3 was used as a cut-height value to obtain more characteristically similar genes **(Figure 2H)**. In addition, all genes in the turquoise module were clustered into a blue module **(Figure 2I)**. Consistent with the aforementioned observation, the light-yellow module was selected for the following analysis **(Figure 2J)**.

### Identification of biological functions and hub genes simultaneously targeted in EAT and the RAA

**Figure 3:**
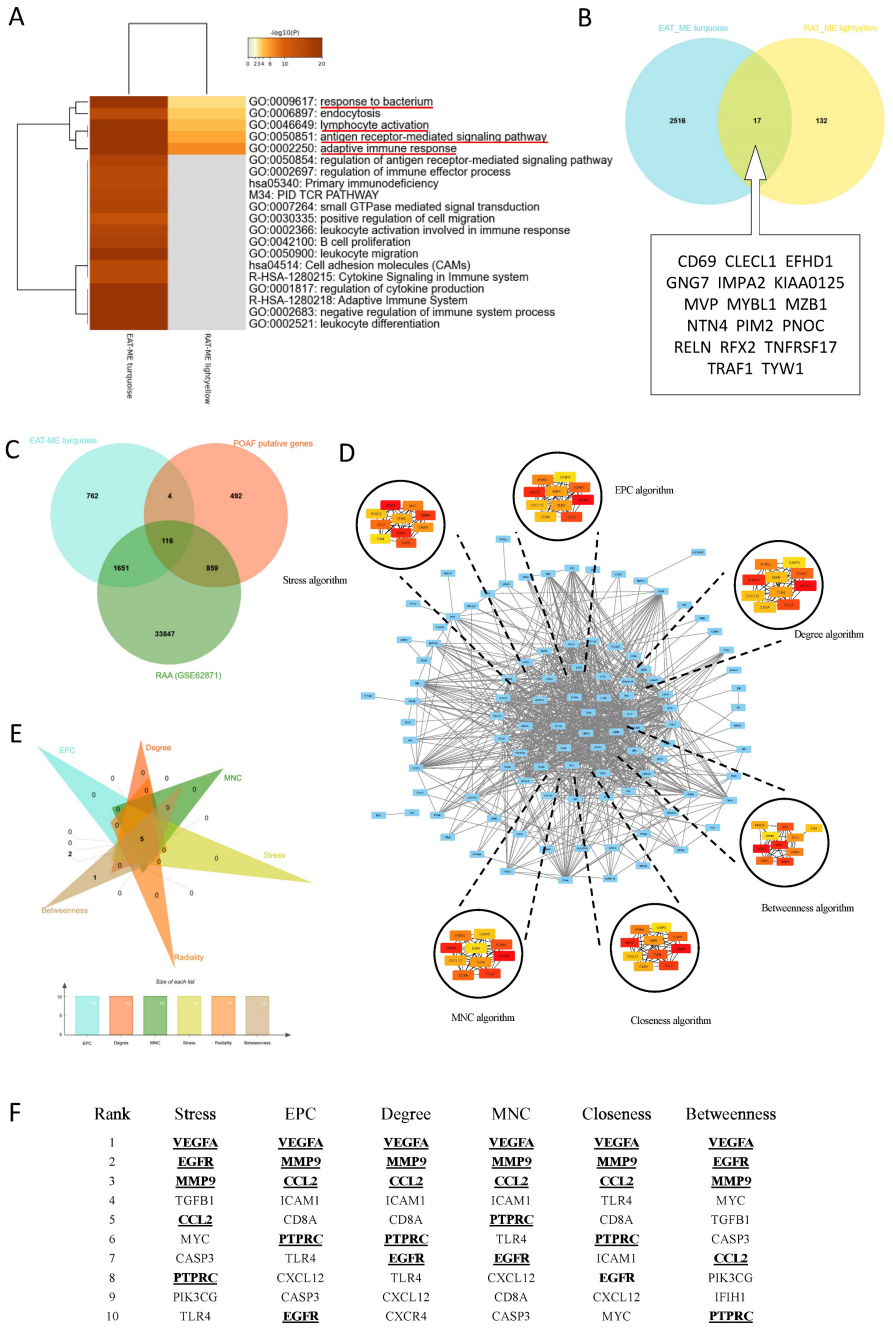
** Identification biological functional and hub genes simultaneously targeted in EAT and the RAA. A.** Pathway enrichment analysis of turquoise and light-yellow modules. Biological functions that were enriched in the top 20 genes with the smallest *p* value. **B.** Venn diagram of the two modules. The 17 genes listed in the box were identified as common genes. **C.** Venn diagram of the POAF putative genes, all genes detected in GSE62871, and all genes obtained in the turquoise module. **D.** Visualization of the protein-protein interaction (PPI) network of the obtained genes from Figure 3C, and top 10 hub genes identified by six algorithms including Stress, EPC, Degree, MNC, Closeness and Betweenness. **E-F.** Venn diagram and table of genes obtained from the above six algorithms. The identified hub genes are indicated with underlining.

To identify biological functions and hub genes simultaneously targeted in EAT and the RAA, both Meatascape and a Venn diagram were used for analysis (**Figure 3A-B**). There were a few genes obtained simultaneously targeted in both EAT and LAA, and limited biological functions are noted to be shared by these two tissue types **(Figure 3A)**. Attractive terms included 'GO:0009617: response to bacterium', 'GO:0046649: lymphocyte activation', 'GO:0050851: antigen receptor-mediated signaling pathway' and 'GO:0002250: adaptive immune response' **(Figure 3A)**. The PPI network could not be constructed due to too few genes being selected in the Venn diagram **(Figure 3B)**. We therefore obtained POAF-associated genes through OMIM and Gene Cards, crossed them with the genes in MEturquoise and all the genes in GSE62871, and then constructed the PPI network using these crossed genes **(Figure 3C)**. The network consisted of 103 nodes and 832 edges **(Figure 3D)**. Six algorithms of CytoHubba, namely Stress, EPC, Degree, Betweenness, Closeness and MNC, were used to identify top ten hub genes in each algorithm **(Figure 3D-E)**. Taking the interaction of top ten hub genes in all algorithms, *VEGFA*, *EGFR*, *MMP9*, *CCL2* and *PTPRC* were assessed as the hub genes in the initiation of POAF that simultaneously target EAT and the RAA **(Figure 3F)**.

### Colchicine or AG-1478 improves short-term survival and reduces the recurrence of AF in LPS-stimulated mice

**Figure 4:**
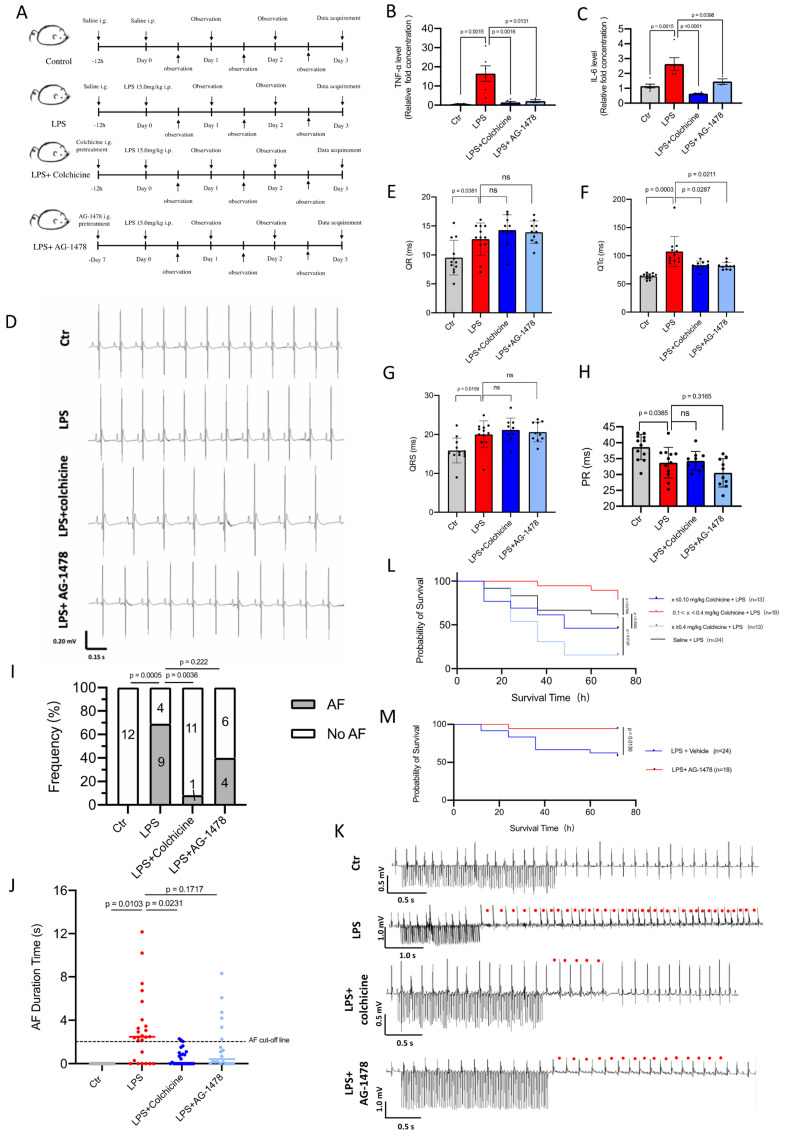
** Colchicine or AG-1478 improves short-term survival and reduces the recurrence of AF in LPS-stimulated mice. A.** Schematic of animal experiments. **B-C.** Bar graphs to compare the TNF-α and IL-6 levels determined by ELISA assay between baseline mice (control group), mice treated with LPS alone (LPS group), mice treated with LPS and colchicine (LPS + colchicine group), and mice treated with LPS and AG-1478 (LPS + AG-1478 group), respectively. n= 3-6 mice. **D.** Representative images of body-surface ECGs for the control group, LPS group, LPS + colchicine group, and LPS + AG-1478 group. **E-H.** Bar graphs to compare QR, QTc, QRS and PR between the control group, LPS group, LPS + colchicine group, and LPS + AG-1478 group. n= 10-12 mice. **I.** Bar graph to compare the number of AF episodes between the control group, LPS group, LPS + colchicine group, and LPS + AG-1478 group. n= 10-13 mice. **J.** Scatter dot plot to compare the AF episode durations between the control group, LPS group, LPS + colchicine group, and LPS + AG-1478 group. n= 10-13 mice. The numbers in bars in Figure 4I represent the number of mice, each mouse had two AF episode durations according to protocol and shown in Figure 4J.** K.** Representative images of intracardiac pacing ECG. Red circles denote the AF events.** L-M.** Survival curves of colchicine or AG-1478 treatment at different doses in LPS-stimulated mice.

To investigate the effects of acute inflammation on cardiac electrophysiology and the validity of pharmacological intervention we designed *in vivo* experiments using mice under the same condition (**Figure 4A**). Consistent with the previous studies^36^, we observed that LPS stimulation promoted the release of inflammation cytokines (**Figure 4B-C**) and disturbed electrocardiographic homeostasis (**Figure 4D**). In surface electrocardiographic analysis, LPS-stimulated mice displayed significantly longer durations of QR waves (**Figure 4E**), QTc waves (**Figure 4F**) and QRS waves (**Figure 4G**) compared to those of control mice, whereas the PR interval was significantly shortened in LPS-stimulated mice (**Figure 4H**). When programmed intracardiac stimulation was applied, LPS-stimulated mice displayed significantly increased number of episodes and episode durations as compared to controls, indicating enhanced AF susceptibility **(Figure 4 I-J)**. The longest duration time of AF in LPS-stimulated mice was 52.0 s **(Figure 4K)**.

Acute inflammation is a major factor that may trigger POAF^2^ and excessive inflammation promotes electrical remodeling and increases susceptibility to POAF^7^. We next selected colchicine, a controversial anti-inflammatory agent, for pharmacological intervention in LPS-stimulated mice **(Figure 4A)**. To validate the identified hub genes, we also selected an EGFR inhibitor (AG-1478) for pharmacological intervention **(Figure 4A)**. Within the effective doses, the administration of colchicine or AG-1478 significantly reduced the LPS-stimulated release of inflammation cytokines (**Figure 4B-C**), and normalized QTc (**Figure 4F**). This observation is in line with previous studies in which the QTc interval is significantly longer in the patients undergoing coronary artery bypass graft (CABG) surgery, and where this could be considered as a predictive value for POAF^37^,^38^. Interestingly, we observed that the dose-response curve of colchicine on the survival rate of LPS-stimulated mice was in a U-shape, with doses between 0.10-0.40 mg/kg greatly improving survival rate of the mice under intense inflammation (*p*= 0.0769). Conversely, either high dose (≥ 0.40 mg/kg, *p*= 0.0126) or low dose (≤0.10 mg/kg, *p*= 0.3583), displayed the opposite effect **(Figure 4L)**. Short-term survival was also significantly improved in LPS-stimulated mice co-administered with AG-1478 at the conventional dose (*p*= 0.013, **Figure 4M**). Moreover, colchicine significantly alleviated the AF burden in LPS-stimulated mice under programmed intracardiac stimulation by reducing the number of episodes **(***p*= 0.0036, **Figure 4I)** and episode durations **(***p*= 0.0231, **Figure 4J)**, whereas administration of AG-1478 alone showed a trend to reduce the occurrence of AF, but with no statistically significant difference in episode number **(***p*= 0.222, **Figure 4I)** or episode time **(***p*= 0.187, **Figure 4J)**.

### Colchicine or AG-1478 ameliorates myeloid cell infiltration and decreases circulating neutrophils in LPS-stimulated mice

**Figure 5:**
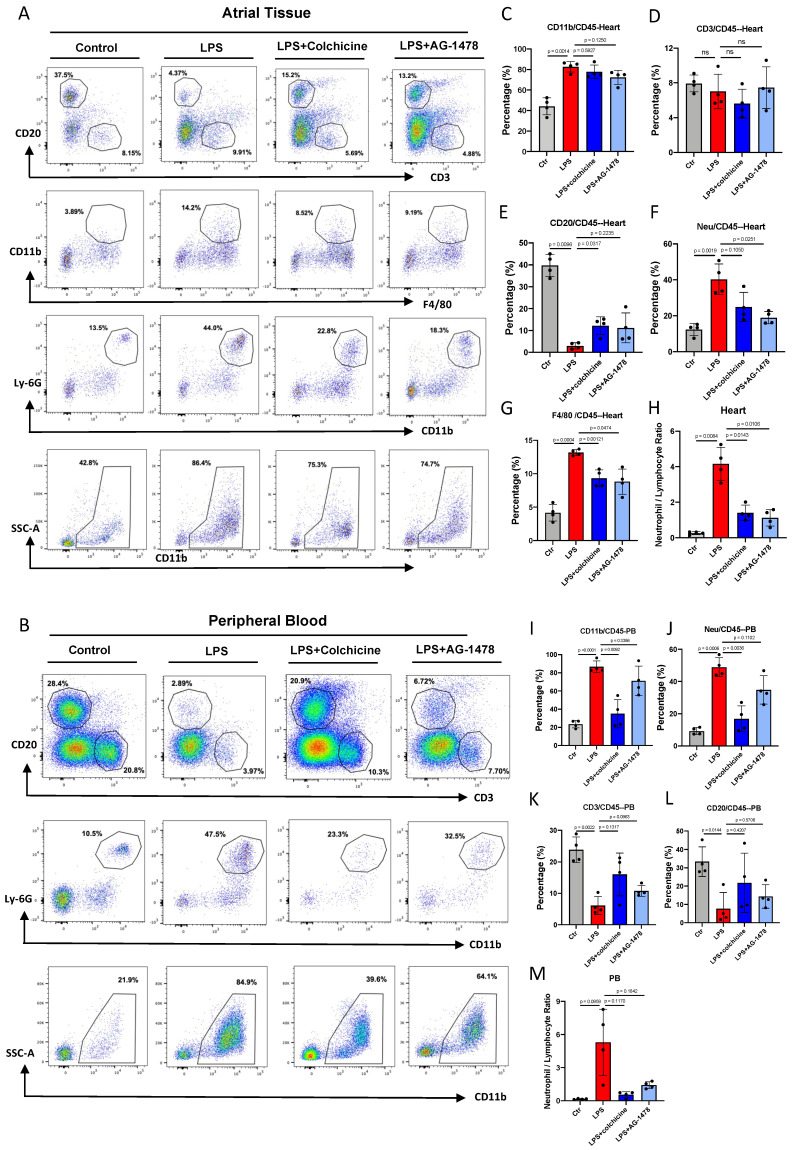
** Colchicine or AG-1478 ameliorates myeloid cell infiltration and decreases circulating neutrophils in LPS-stimulated mice. A.** Proportions of infiltration of inflammatory cells as analyzed by FACS in atrial tissues of the control group, LPS group, LPS + colchicine group, and LPS + AG-1478 group. **B.** Proportions of circulating inflammatory cells as analyzed by FACS in peripheral blood (PB) of the control group, LPS group, LPS + colchicine group, and LPS + AG-1478 group. **C-H.** Bar graphs to compare the proportions of myeloid cells, T cells, B cells, neutrophils, macrophages, and neutrophil/lymphocyte ratio (NLR) in atrial tissues of the control group, LPS group, LPS + colchicine group, and LPS + AG-1478 group. n= 4 mice. **I-M.** Bar graphs to compare proportions of myeloid cells, T cells, B cells, neutrophils, and NLR in the PB of the control group, LPS group, LPS + colchicine group, and LPS + AG-1478 group. n= 4 mice.

To determine the most activated type of immune cells to migrate and infiltrate atrial tissues under acute inflammation, and also to validate the pharmacological intervention, we performed flow cytometry analysis in atrial tissues and peripheral blood (PB). We observed that the proportions of different immune cells were altered in both atrial tissues and PB in the LPS group, as compared to baseline **(Figure 5A-B)**. In the analysis of atrial tissues, we observed that the proportion of myeloid cells (CD45^+^CD11b^+^) were significantly higher under LPS stimulation **(***p*= 0.0014, **Figure 5C)**, and the proportion of lymphocyte cells correspondingly decreased **(Figure 5D-E)**.

No significant change was observed in the proportion of CD3^+^ T lymphocytes between baseline and LPS groups **(Figure 5D)**. A markedly reduced proportion of CD20^+^ B lymphocytes was observed in the LPS group **(***p*= 0.0096, **Figure 5E)**. The proportions of cells in the myeloid lineage, such as neutrophils **(***p*= 0.0019, **Figure 5F)** and macrophages **(***p*= 0.0004, **Figure 5G),** were significantly increased in response to inflammatory stimulation. Massive myeloid cells accounted for the majority of infiltrating cells **(Figure 5F-G)**. Moreover, the neutrophil/lymphocyte ratio (NLR) was greatly increased at the acute course of inflammation, which was closely related to the AF recurrence/occurrence **(***p*= 0.0084, **Figure 5H)**.

In the analysis of PB, we observed that the proportion of CD11b^+^ cells **(***p*< 0.0001, **Figure 5I)**, especially neutrophils **(***p*= 0.0006,** Figure 5J)** was dramatically increased in the LPS group. By contrast, the proportions of CD3^+^ T lymphocytes and CD20^+^ B lymphocytes exhibited a marked reduction after LPS stimulation **(Figure 5K-L)**. Consistent with the observation in atrial tissues, the NLR was increased in the PB of the LPS group **(***p*= 0.0959,** Figure 5M)**. These results indicate that acute inflammation induces the production and mobilization of myeloid cells.

In atrial tissues, administration of colchicine or AG-1478 in the LPS group greatly reduced the infiltration of macrophages and neutrophils, also reversing the reduction of CD20^+^ B lymphocytes **(Figure 5E-G)**. However, it showed minimal effect on the proportions of myeloid cells and CD3^+^ T lymphocytes **(Figure 5D)**. In peripheral blood, administration of colchicine but not AG-1478, greatly decreased the proportion of the circulating myeloid cells and neutrophils in the LPS group** (Figure 5I-J)**. We also observed that the administration of colchicine or AG-1478 in the LPS group resulted in an increased trend of lymphocyte proportion, but without statistical significance **(Figure 5K-L)**. Importantly, the increased NLR in both atrial tissues and PB of the LPS group was significantly reversed by colchicine or AG-1478 **(Figure 5H and 5M)**. These results demonstrate that colchicine or AG-1478 ameliorates myeloid cell infiltration and decreases circulating neutrophils in PB.

### Colchicine or AG-1478 reduces inflammatory cell infiltration and fibrosis in the atrial tissues of LPS-stimulated mice

**Figure 6:**
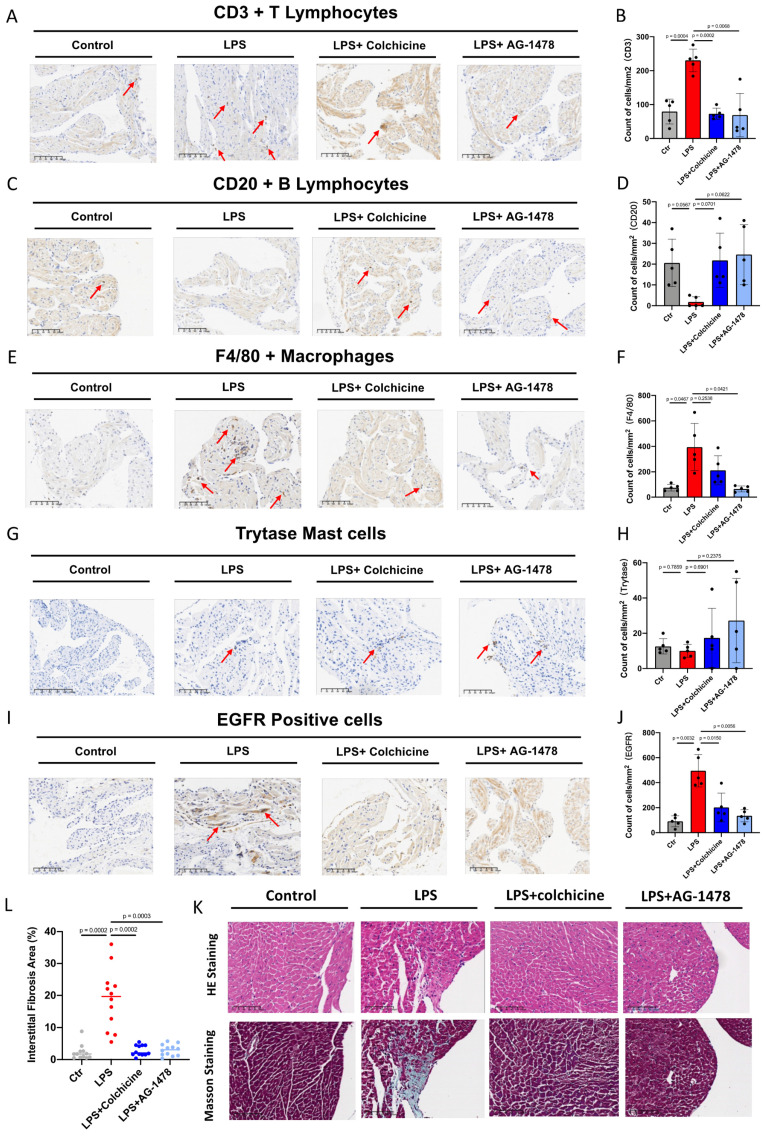
** Colchicine or AG-1478 reduces the inflammatory cell infiltration and fibrosis in atrial tissues of LPS-stimulated mice. A-J.** Immunohistochemical staining and quantification of CD3^+^ T lymphocytes, CD20^+^ B lymphocytes, F4/80^+^ macrophages, mast cells and EGFR positive cells in atrial tissues of the control group, LPS group, LPS + colchicine group, and LPS + AG-1478 group. n= 5 mice. **K.** Representative images of HE and Masson staining on the atrial sections of the control group, LPS group, LPS + colchicine group, and LPS + AG-1478 group. Scale bar, 100 μm. n= 4 mice. **L.** Scatter dot plot to compare the percentage of interstitial fibrosis area in atrial tissues of the control group, LPS group, LPS + colchicine group, and LPS + AG-1478 group. n= 4 mice. Three fields of views were measured for each mouse.

To classify and quantify the infiltrated inflammatory cells, we performed immunochemistry and histology analysis in atrial tissues of LPS-stimulated mice. We observed that number of CD3^+^ T lymphocytes or F4/80 positive macrophages had significantly increased in the LPS group, whereas number of CD20^+^ T lymphocytes had significantly decreased, when compared to baseline **(Figure 6A-F).** Moreover, an antibody against mast cell tryptase was used to detect mast cells. No significant change was observed in the total number of mast cells between baseline and LPS groups **(Figure 6G-H)**. We also found that the expression of EGFR, one of the identified hub genes, was significantly elevated under acute inflammation **(Figure 6I-J)**. Importantly, the aforementioned phenotype of inflammatory cell infiltration, as induced by LPS stimulation, was significantly rescued by the administration of colchicine or AG-1478 **(Figure 6A-J)**. Given that cardiac inflammation is usually accompanied with increased fibrosis, we next assessed the extent of fibrosis in atrial tissues. Compared to baseline, the LPS group demonstrated severe myocardial injury with disorganized and misaligned atrial myocytes, massive inflammatory cell infiltration, and significantly increased fibrosis as evidenced by HE and Masson staining. These too were effectively rescued by colchicine or AG-1478 **(Figure 6K-L)**.

### Colchicine or AG-1478 restores the altered expression of ion channels, Ca^2+^-handling proteins and α-tubulin in atrial tissues of LPS-stimulated mice

**Figure 7:**
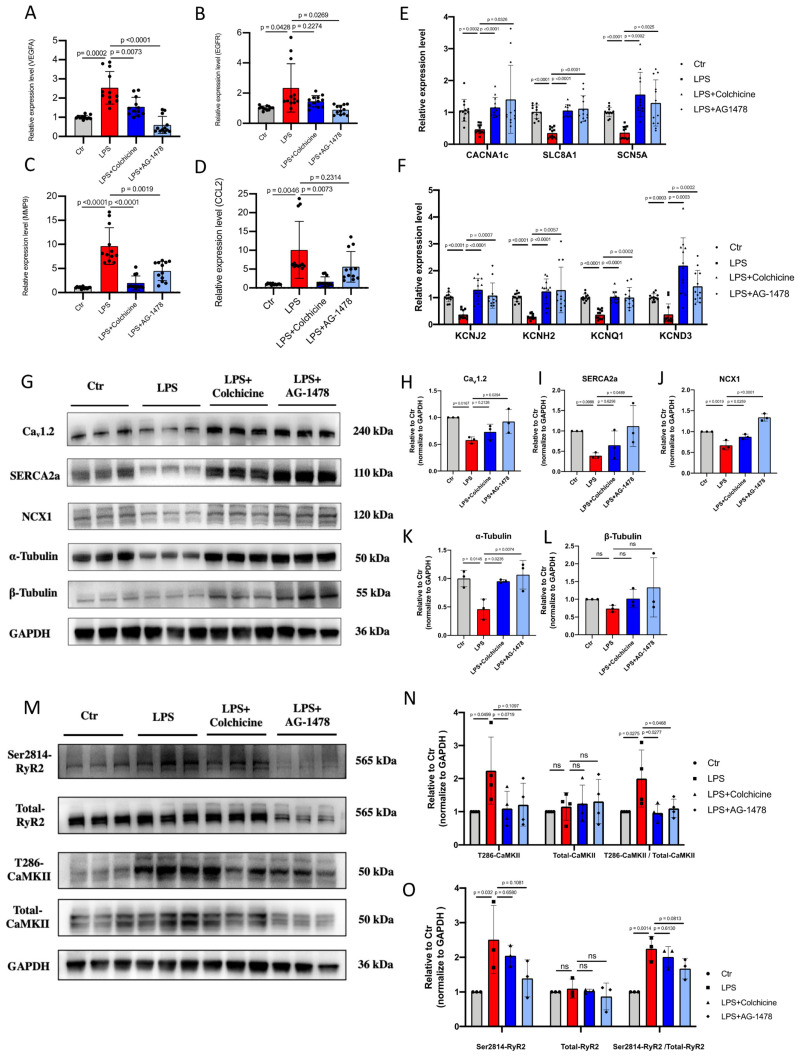
** Colchicine or AG-1478 restores the altered expression of ion channels, Ca^2+^-handling proteins and α-tubulin in atrial tissues of LPS-stimulated mice. A-D.** Bar graphs to compare the mRNA expression levels of hub genes (*VEGFA*, *EGFR*, *MMP9*, *CCL2*) between atrial tissues of the control group, LPS group, LPS + colchicine group, and LPS + AG-1478 group. n= 12 mice. **E-F.** Bar graphs to compare the mRNA expression levels of multiple ion channel genes (*CACNA1C*, *SLC8A1*, *SCN5A*, *KCNJ2*, *KCNH2*, *KCNQ1* and *KCND3*) between the atrial tissues of the control group, LPS group, LPS + colchicine group, and LPS + AG-1478 group. n= 12 mice. **G.** Western blot analysis of the protein expression of Ca_v_1.2, NCX1, SERCA2a, α-Tubulin and β-Tubulin in atrial tissues of the control group, LPS group, LPS + colchicine group, and LPS + AG-1478 group. **H-L.** Bar graphs to compare the protein expression of Ca_v_1.2, NCX1, SERCA2a, α-Tubulin and β-Tubulin in different groups in Figure 7G. n= 3 independent experiments. **M.** Western blot analysis of the protein expression of Ser2814-RyR2, total RyR2, T286-CaMKII and total CaMKII in atrial tissues of the control group, LPS group, LPS + colchicine group, and LPS + AG-1478 group. **N-O.** Bar graphs to compare the protein expression of Ser2814-RyR2, total RyR2, T286-CaMKII and total CaMKII in different groups in Figure 7M. n= 3-4 independent experiments.

All identified hub genes were upregulated in LPS-stimulated mice **(Figure 7A-D)**. Meanwhile, consistent with our histological observation, mRNA expression of *EGFR* was significantly increased in the atrial tissues of LPS-stimulated mice as compared to baseline **(Figure 7A)**. One recent study has shown that inhibition of EGFR impairs fungal-triggered immune responses, for which a Ca^2+^ influx is essential^39^. EGFR is actively involved in Ca^2+^ ion transport, Ca^2+^ channel activity and Ca^2+^ ion transmembrane transport^40^. Therefore, we sought to investigate if the elevation of *EGFR* expression may lead to corresponding changes in the expression of ion channels and Ca^2+^-handling proteins in atrial tissues of LPS-stimulated mice. Electrical remodeling promotes alteration of locations and densities of different ion channels in atrial tissues^41^.

We consistently observed that mRNA expression levels of a panel of ion-channel genes were all significantly reduced in the LPS group as compared to baseline, suggesting the occurrence of ion channel remodeling **(Figure 7E-F)**. Moreover, Western blot analysis revealed significantly reduced expression of Ca^2+^-handling proteins in the LPS group, including Ca_v_1.2, NCX1 and SERCA2a **(Figure 7G-J and Supplemental Figure 2)**. The cytoskeleton is known as one of the therapeutic targets of AF^42^-^44^. Previous studies reported that derailment of α-tubulin proteostasis contributes to the occurrence of AF^43^,^44^. Interestingly, the protein expression level of α-tubulin was significantly lower in LPS group than that in baseline, whereas expression levels of β-tubulin were comparable **(Figure 7K-L and Supplemental Figure 2)**. Administration of colchicine or AG-1478 greatly restored the reduced expression of ion channels, Ca^2+^-handling proteins and α-tubulin seen in LPS group **(Figure 7E-L and Supplemental Figure 2)**.

It has also been reported that pre-existing Ca^2+^-handling abnormalities and CaMKⅡ/RyR2 signaling are evident in cardiomyocytes from POAF patients^2^. We further assessed the protein expression levels of CaMKII and RyR2 by Western blot **(Figure 7M and Supplemental Figure 3)**. Total expression levels of CaMKII and RyR2 were unchanged between baseline and LPS groups **(Figure 7N-O and Supplemental Figure 3)**. However, the expression level of phosphorylated CaMKII at T286 site was significantly higher in the LPS group than that in baseline, which was significantly restored by colchicine or AG-1478 **(Figure 7N and Supplemental Figure 3)**. We also observed significantly enhanced CaMKII-dependent phosphorylation of RyR2 at S2814 site in LPS group, which was partially restored by colchicine or AG-1478 **(Figure 7O and Supplemental Figure 3)**.

### Colchicine or AG-1478 protects against cell death and stabilizes microtubules from acute inflammation in iPSC-aCMs

**Figure 8:**
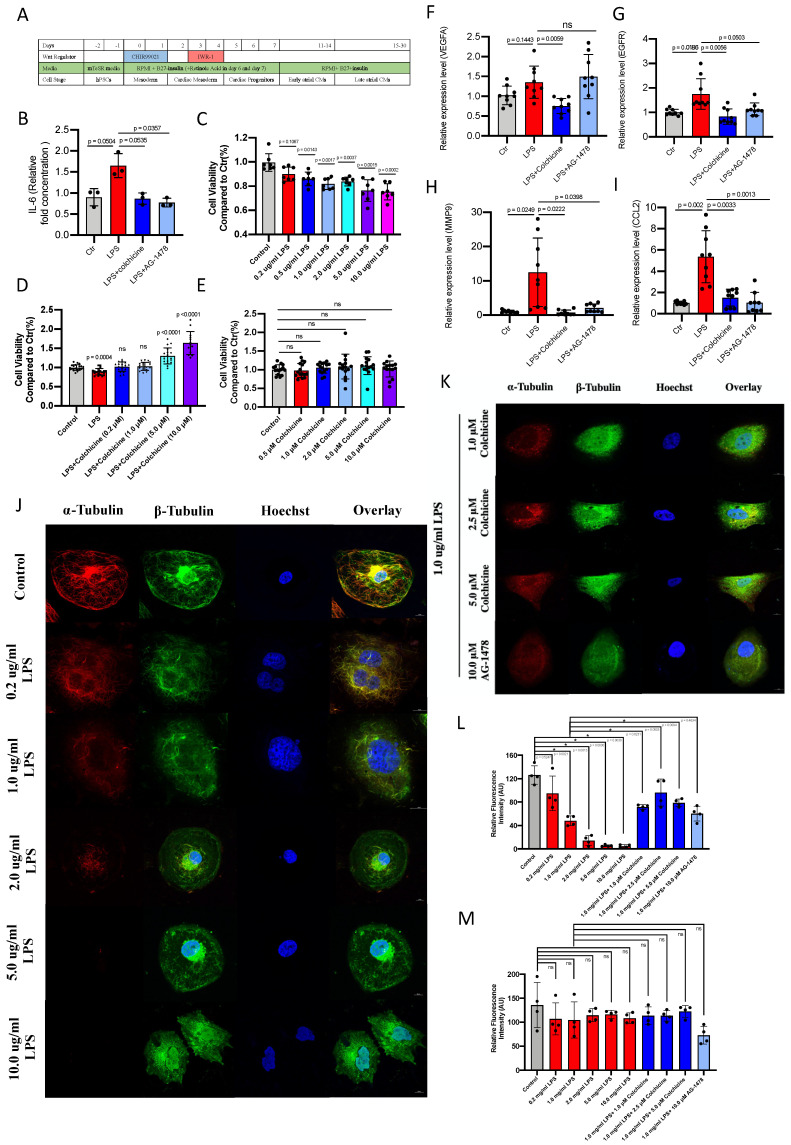
** Colchicine or AG-1478 protects against cell death and stabilizes microtubules from acute inflammation in iPSC-aCMs. A.** Protocol for generation of iPSC-aCMs. **B.** Bar graph to compare IL-6 levels as determined by ELISA assays between baseline iPSC-aCMs (control), iPSC-aCMs treated with LPS alone (LPS group), iPSC-aCMs treated with LPS and colchicine (LPS + colchicine group), and iPSC-aCMs treated with LPS and AG-1478 (LPS + AG-1478 group), respectively. n= 3 biological replicates. **C.** Bar graph to compare cell viability as detected by CCK-8 assays between iPSC-aCMs treated with LPS at different concentrations for 24 hours. n= 7 biological replicates. **D.** Bar graph to compare cell viability between iPSC-aCMs treated with 1.0 μg/ml LPS and colchicine at different concentrations for 24 hours. n= 13-21 biological replicates. **E.** Bar graph to compare cell viability between iPSC-aCMs treated with colchicine at different concentrations for 24 hours. n= 15 biological replicates. **F-I.** Bar graphs to compare mRNA expression levels of hub genes (*VEGFA*, *EGFR*, *MMP9*, *CCL2*) between the control group, LPS group, LPS + colchicine group, and LPS + AG-1478 group. n= 9 biological replicates. **J-K.** Representative immunofluorescence staining of α-Tubulin (red) and β-Tubulin (green) in baseline iPSC-aCMs, iPSC-aCMs treated with LPS at different concentrations for 24 hours; iPSC-aCMs treated with 1.0 μg/ml LPS and colchicine at different concentrations for 24 hours; and iPSC-aCMs treated with 1.0 μg/ml LPS and 10 μM AG-1478. Scale bar, 10 μm.** L-M.** Bar graphs to compare the fluorescence intensity of α-Tubulin and β-Tubulin between different groups in J-K. The stained microtubules from 4 images were selected and quantified by ImageJ.

We next employed iPSC-aCMs to investigate whether the electrophysiological and immunological alterations under acute inflammation in mice could be recapitulated in a human-based *in vitro* model. The iPSCs were differentiated into atrial-like myocytes using a 2D differentiation protocol **(Figure 8A and Supplemental Figure 4)**. Immunofluorescence demonstrated that the generated iPSC-aCMs were stained positive for the atrial-specific markers MLC2a and NR2F2 **Supplemental Figure 5A-B)**. Single-cell patch clamp analysis revealed a characteristic action potential profile in the iPSC-aCMs **(Supplemental Figure 5C-E)**. We observed a significantly increased amount of IL-6 in LPS-stimulated iPSC-aCMs as compared to baseline, which was restored by the administration of colchicine or AG-1478 **(Figure 8B)**. LPS stimulation caused significantly reduced cell viability in iPSC-aCMs in a concentration-dependent manner **(Figure 8C)**. Treatment of colchicine at escalating concentrations rescued the cell death induced by LPS stimulation in iPSC-aCMs **(Figure 8D)**, whereas colchicine alone had minimal effect on cell viability **(Figure 8E)**. Consistent with the observations in atrial tissues of LPS-stimulated mice, mRNA expression levels of the four hub genes (*VEGFA*, *EGFR*, *MMP9* and *CCL2*) were significantly higher in LPS-stimulated iPSC-aCMs than those in the baseline, and these were restored by colchicine or AG-1478 treatment **(Figure 8F-I)**. Likewise, we observed that the expression of α-tubulin was significantly diminished in iPSC-aCMs with LPS stimulation at different concentrations, which was rescued by colchicine or AG-1478 **(Figure 8J-L)**. The expression of β-tubulin remained unchanged between the different groups **(Figure 8J-K and 8M)**. Moreover, LPS stimulation caused a disorganized microtubule network in iPSC-aCMs starting at a low concentration (0.20 μg/ml), with most of the normal tubular-like structures disappearing upon the concentration of LPS reaching 1.0 μg/ml **(Figure 8J)**. Spot-like β-tubulin signals, as indicated by green fluorescence, were only seen in colchicine-treated iPSC-aCMs **(Figure 8K)**. No significant morphological changes in myofilaments were observed in different groups, suggesting that LPS stimulation or colchicine treatment has no effect on myofilaments** (Supplemental Figure 6-7)**.

### Colchicine or AG-1478 normalizes the prolonged field potential duration in LPS-stimulated iPSC-aCMs

**Figure 9:**
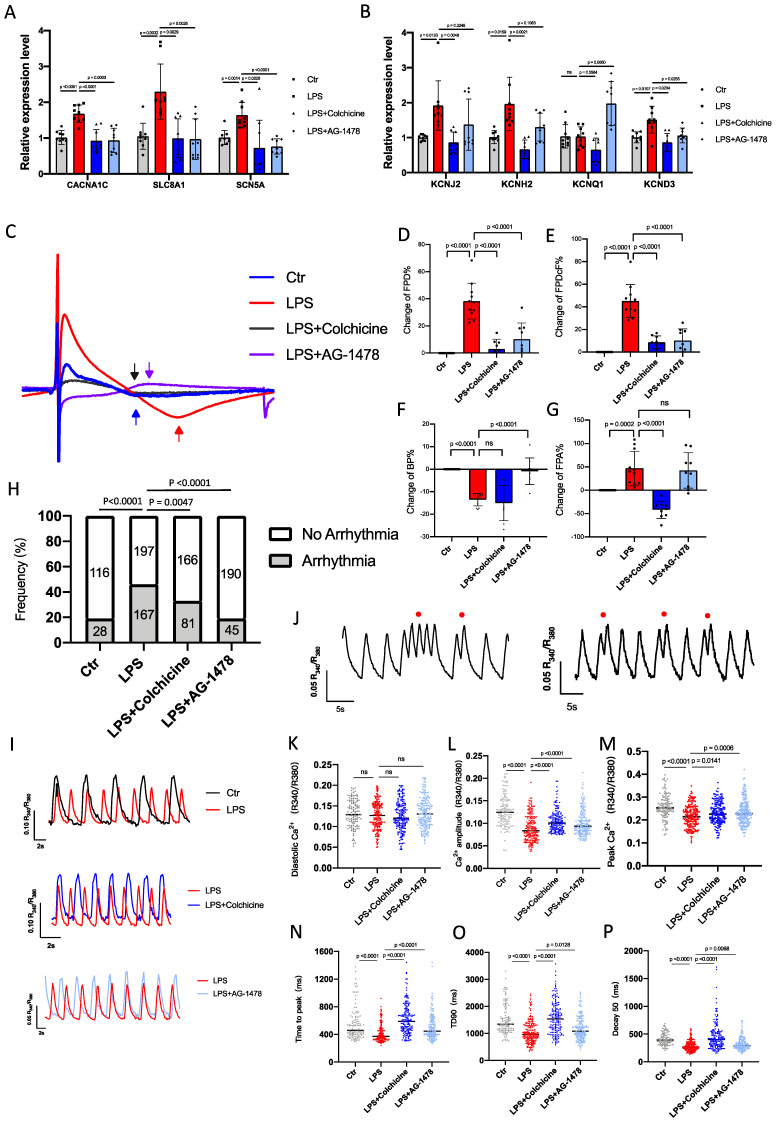
** Colchicine or AG-1478 normalizes the prolonged field potential duration in LPS-stimulated iPSC-aCMs. A-B.** Bar graphs to compare the mRNA expression levels of multiple ion channel genes (*CACNA1C*, *SLC8A1*, *SCN5A*, *KCNJ2*, *KCNH2*, *KCNQ1* and *KCND3*) between iPSC-aCMs of the control group, LPS group, LPS + colchicine group, and LPS + AG-1478 group. n= 9 biological replicates. **C.** Representative field potential tracings recorded from iPSC-aCMs of the control group, LPS group, LPS + colchicine group, and LPS + AG-1478 group. The iPSC-aCMs were derived from two healthy control iPSC lines. **D-G.** Bar graphs to compare the change of FPD%, change of FPDcF%, change of BP% and change of FPA% between different groups in Figure 9C. n= 9-11 biological replicates. **H.** Bar graph to compare the percentage of arrhythmia‐like irregular Ca^2+^ transients between iPSC-aCMs of the control group, LPS group, LPS + colchicine group, and LPS + AG-1478 group. n= 116-198 cells. **I.** Representative Ca^2+^ transient tracings recorded from iPSC-aCMs of the control group, LPS group, LPS + colchicine group, and LPS + AG-1478 group.** J.** Representative Ca^2+^ transient tracings recorded from iPSC-aCMs of LPS group. Red circles denote arrhythmia‐like irregular Ca^2+^ transients. **K-P.** Scatter dot plots to compare diastolic [Ca^2+^]_i_, Ca^2+^ amplitude, peak Ca^2+^, time to peak, transient duration 90 (TD90) and decay50 between the different groups of Figure 9I.

To investigate the molecular mechanisms underlying the alteration of the electrical properties in mice under LPS stimulation, we assessed the effects of acute inflammation (1.0 μg/ml LPS) on the electrophysiological characteristics in iPSC-aCMs. qPCR analysis revealed that mRNA expression levels of multiple ion channel genes, including *CACNA1C*, *SLC8A1*, *SCN5A*, *KCNJ2*, *KCNH2*, *KCNQ1* and *KCND3*, were significantly higher in LPS-stimulated iPSC-aCMs than those in baseline, which were largely restored by colchicine or AG-1478 treatment** (Figure 9A-B)**. To characterize electrophysiological properties, field potentials were recorded from iPSC-aCMs by MEA and key parameters were quantified **(Figure 9C)**.

We observed that FPD and FPDc were significantly prolonged in LPS-stimulated iPSC-aCMs compared to the baseline **(Figure 9D-E)**. Consistent with the observations in mice, the prolonged FPD/FPDc phenotype was effectively rescued by treatment of colchicine or AG-1478 **(Figure 9D-E)**. Moreover, LPS stimulation significantly increased the beating rate, which was eased by AG-1478 **(Figure 9F)**. FPA was also dramatically increased under LPS stimulation, which was reversed by the administration of colchicine **(Figure 9G)**.

### Colchicine or AG-1478 rescues the arrhythmic phenotype and restores Ca^2+^ homeostasis in LPS-stimulated iPSC-aCMs

To test whether cardiomyocyte-restricted inflammation is sufficient to increase arrhythmias, iPSC-aCMs were incubated with 1.0 μg/ml LPS for 24 hours, and Ca^2+^ imaging was conducted using fura‐2 AM dye to ratiometrically record Ca^2+^ transients. As expected, baseline iPSC-aCMs showed a regular Ca^2+^ transient profile **(Figure 9H-I)**. However, LPS stimulation caused significantly increased proportions of arrhythmia-like irregular Ca^2+^ transients in iPSC‐aCMs **(Figure 9H and 9J)**. Diastolic Ca^2+^ levels were comparable between baseline and LPS-stimulated iPSC-aCMs **(Figure 9K)**, whereas Ca^2+^ amplitude and peak Ca^2+^ were both significantly reduced in LPS-stimulated iPSC-aCMs **(Figure 9L-M)**. LPS stimulation also caused significantly shortened time to peak, transient duration 90 (TD90), and decay 90, in iPSC‐aCMs** (Figure 9N-P)**. Importantly, treatment of colchicine or AG-1478 in LPS-stimulated iPSC-aCMs greatly mitigated the Ca^2+^ transient irregularities and restored the Ca^2+^ transient properties **(Figure 9H-P)**.

## Discussion

In this study, using a mouse model, we have verified the association between inflammation and POAF and discovered corresponding immunological alterations under acute inflammation. In accordance with previous reports, POAF is evoked when transient peri-operative triggers interact with pre-existing arrhythmogenic substrates(1. Local and systemic inflammation accounts for a large proportion of these substrates^2^,^3^,^6^. EAT has been suggested as a source of inflammatory mediators and a producer of pro-inflammatory adipokines^9^,^10^. Moreover, adipocytes from EAT have been reported to invade adjacent structures, with the infiltration of contiguous atrial myocardium conferring atrial conduction abnormalities^11^.

Catheter ablation, atriotomy, and pericardiotomy often results in acute inflammation and activates an immune response. The activated immune cells play various different roles in the initiation of AF^8^. Neutrophils occupy a large proportion of immune cells in patients undergoing surgery, and these are believed to be the main source of reactive oxygen species (ROS) and myeloperoxidase (MPO)^1^,^7^. Macrophages exert their effects by releasing cytokines, leading to both structural and electric atrial remodeling in AF^8^. Mast cells have also been confirmed as a contributors to the progression of fibrosis in AF, as mediated by platelet-derived growth factor A 34. However, T and B lymphocytes are adaptive immune cells and few studies have been conducted related to lymphocytes and AF^8^. In this study, we used the CIBERSORT algorithm to identify altered immune cells, which were then verified using FACS and IHC staining. Consistent with the metascape results, we found only T lymphocytes, macrophages and neutrophils to be candidates for playing important roles in the occurrence of POAF. POAF is generally a transient phenomenon with peaking incidence at 2-4 days after surgery. Inflammation, cytokines, and the autonomic nervous system are the major triggers which act to increase vulnerability and initiate POAF^1^. Increased neutrophilia represents activated non-specific inflammation whereas lymphopenia is considered to be more of an indicator of poor general heath and physiological stress^45^. NLR integrates these two important and opposite immune pathways, and is therefore considered a measure of both systemic inflammation and stress response^46^. Recent evidence has shown that, whether measured at baseline or post-surgery, a high NLR is associated with increased risk of AF occurrence/recurrence^46^. In our study, the significant increases in the NLR, as observed in both PB and atrial tissues, could be relieved upon the administration of anti-inflammatory agents.

In addition to immunological alterations, inflammation can promote atrial electrical remodeling and increased the propensity for developing arrhythmias both *in vitro* and *in vivo*. Mice under intensive inflammation had a longer duration of QR, QTc and QRS waves, but a shortened PR intervals compared to controls. The prolongation of QTc could be reversed by the treatment of colchicine or AG-1478. Strikingly, QT and QTc intervals were significantly longer in the patients undergoing CABG surgery, and these could be considered as a predictive measure for POAF^37^,^38^. After programmed intracardiac stimulation, AF susceptibility was significantly increased under inflammation status, with significantly increased number of episodes and increased episode durations after LPS stimulation. To exclude immune cells from pathogenesis, we sought to investigate the effect of cardiomyocyte-specific inflammation in electrical remodeling^47^. By employing a human iPSC-aCM model, we found that cardiomyocyte-specific activation of inflammation enhanced arrhythmic susceptibility, prolonged FPD, and perturbed Ca^2+^ fluxes. In accordance with the previous study^2^, pre-existing Ca^2+^ handling abnormalities and imbalance in Ca^2+^-handling proteins promoted the occurrence of POAF under exposure to inflammatory mediators. We found that expression levels of Ca^2+^-handling proteins were significantly changed in both LPS-stimulated mice and iPSC-aCMs. Furthermore, we showed that inflammation enhanced the expression of phosphorylated CaMKII and RyR2 in mice, which was consistent with the previous report^2^. EGFR is a protein tyrosine kinase (PTK) and is involved in important cellular processes such as cellular proliferation, differentiation and electrophysiology, via the tyrosine phosphorylation of cardiac ion channels^40^. Cardiac ion channels, including L-type Ca^2+^ channel^48^ and K^+^ channels^49^, are regulated by PTKs, and the voltage-gated Na^+^ current (I_Na_) is upregulated by activation of EGFR kinase in ventricular myocytes^50^. Screening of genetic variants by GWAS revealed *EGFR* and *RYR2* as central regulators of Ca^2+^ signaling related to the progression and recurrence of AF^51^. POAF is not just a monogenic disease, it is considered as a multifactorial disease with a strong genetic background^1^. Pharmacological inhibition of EGFR by AG-1478 did not achieve the desired results in our study, particularly when compared to colchicine treatment which was able to suppress all of the hub genes.

Colchicine is one of the oldest remedies that is still in use today^16^. Recently, much attention has been focused upon colchicine in patients with coronary disease^52^,^53^. Emerging evidence has promoted considerable debate regarding the efficacy and safety of colchicine towards preventing POAF 17,54,55. Colchicine is recognized to have a narrow therapeutic margin, with only concentrations of between 0.015 and 0.030 mg/kg, bringing clinical benefits^56^. Gastrointestinal symptoms are its predominant side effects. With concentrations between 0.030 and 0.50 mg/kg, patients often present gastrointestinal symptoms such as vomiting and diarrhea and with concentrations of greater than 0.50 mg/kg producing higher fatality rates^56^,^57^. Overall, longer administrations and higher doses of colchicine exacerbate such side effects. The half-life of colchicine is about 27-31 hours and acute biological effects require 24-48 hours to fully develop^16^. In the evaluation of prevention, various doses of colchicine were administrated 12 hours before LPS stimulation. Notably, we found that the dose-response curve of colchicine on the survival rate of LPS-stimulated mice was in a U-shape, in which the dose within 0.10-0.40 mg/kg greatly improved survival rate of the mice under intense inflammation, whereas high or low doses had the opposite effect. In iPSC-aCM model, treatment of colchicine reduced cell death in a concentration-dependent manner under inflammation, whereas colchicine alone had no effect on cell death. The clinical benefit of colchicine that we obtained in mice was only when it was used between 0.10~0.40 mg/kg. According to body surface area between different species, we calculated the human-equivalent dose (HED) by dividing the rat dose using 12.3^58^. Interestingly, when the dose adjustment of colchicine was calculated as standard weight (60.0-75.0 kg), our result was similar to that of its recommended clinical usage (0.5mg per day)^52^. However, the colchicine administration in our study was only intended for one time use, before LPS stimulation. Repeated colchicine administration might aggravate adverse reactions, and attention should be paid to cardiac condition of postoperative patients. Prior identification of the specific benefit patients may derive from colchicine remains a key issue. The cytoskeleton is known as one of the therapeutic targets of AF^42^-^44^. Previous studies have reported that derailment of α-tubulin proteostasis contributes to the occurrence of AF^43^,^44^. In our study, we found that the microtubule network was similarly affected by inflammation in a concentration-dependent manner. The polymerization state of microtubules regulates the dynamics of cardiac K^+^ channels and powerfully influences cardiac electrical activity^59^. In our observations, inflammation produced a breakdown of the microtubule network and promoted α-tubulin degradation. The treatment of colchicine stabilized β-tubulin and reversed α-tubulin degradation, whereas the EGFR inhibitor AG-1478 had a minimal effect on the microtubule network.

This study has several limitations. Firstly, it is difficult to evaluate the degree of inflammation and volume of EAT in mice. This hampered our direct assessment of anti-inflammatory drugs on EAT. We can only speculate on their effectiveness by outcome events (AF or not). Also, we may not have sufficiently captured the proportion of EAT-derived inflammation in recurrences of AF. Secondly, we investigated the alterations of cardiac electrophysiology under acute inflammation *in vivo* and *in vitro*. Most observations between *in vivo* and *in vitro* studies were similar, with only a few differences. The *in vitro* studies using iPSC-aCMs focused on inflammation from cardiomyocyte-specific activation. It is undeniable that either immune cells themselves or the crosstalk between immune cells and the myocardium, could alter electrical properties. Though it would be of interest to investigate such a factor, it would be difficult to determine alterations of electrophysiology by co-culturing all immune cells with iPSC-aCMs.

## Conclusions

**Figure 10:**
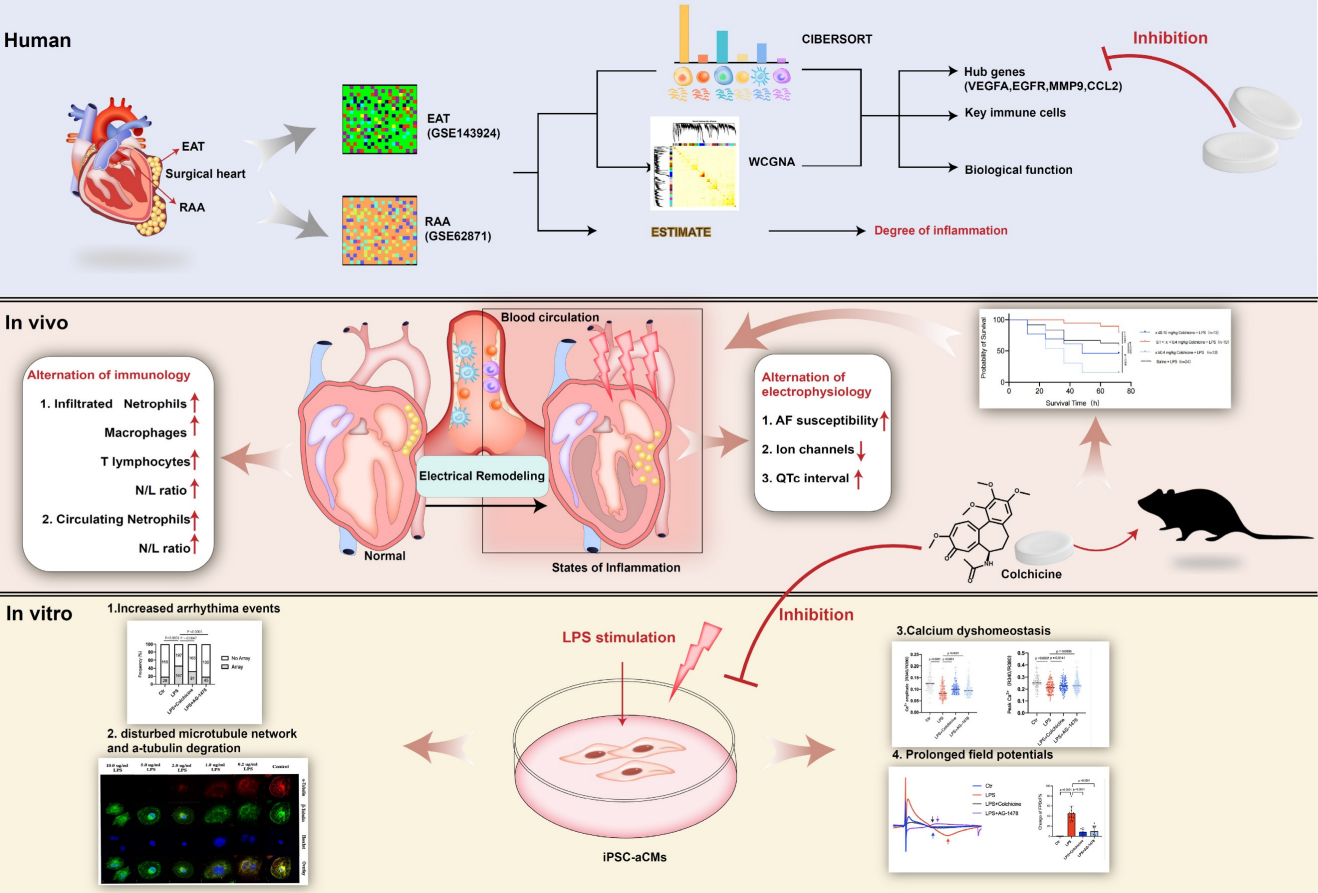
** Take-home message.** We identify *VEGFA*, *EGFR*, *MMP9* and *CCL2* as hub genes simultaneously targeted in epicardial adipose tissue (EAT) and the right atrial appendage (RAA) by performing bioinformatic analysis. Lipopolysaccharide (LPS)-stimulated mice demonstrate electrical remodeling, enhanced atrial fibrillation (AF) susceptibility, immune cell activation, inflammatory infiltration, and fibrosis. Stimulation of LPS in human induced pluripotent stem cell-atrial cardiomyocytes (iPSC-aCMs) causes arrhythmias, abnormal Ca^2+^ signaling, reduced cell viability, disrupted microtubule networks, and increased α-tubulin degradation. Colchicine, considered as a controversial anti-inflammatory agent for in clinical practice, attenuates electrical remodeling of POAF through inhibition of immune-related hub genes and stabilization of microtubules.

Our study provides evidence that EAT-derived inflammation contributes to AF recurrence after surgery. Anti-inflammatory drugs simultaneously targeting EAT and atrial tissues have considerable potential towards the prevention of POAF. Among these we highlight colchicine administration as effective in POAF prevention, and attention should be paid to individual differences and dose adjustments (**Figure 10**).

## Supplementary Material

Supplementary figures and tables.

## Abbreviations

POAF: post-operative atrial fibrillation
AF: atrial fibrillation
EAT: epicardial adipose tissue
LPS: lipopolysaccharide
NS: normal saline
DMSO: dimethyl sulfoxide
ELISA: enzyme linked immunosorbent assay
PB: peripheral blood
SR: sinus rhythm
RAA: right atrial appendage
ECG: electrocardiogram
PFA: paraformaldehyde
HE: hematoxylin-eosin
IHC: immunohistochemistry
iPSCs: induced pluripotent stem cells
ALP: alkaline phosphatase
iPSC-aCMs: iPSC-derived atrial cardiomyocytes
MEA: multi-electrode array
BP: beating period
FPD: field potential duration
FPA: field potential amplitude
ISI: inter-spike interval
RIPA: radioimmunoprecipitation assay
cDNA: complementary DNA
