# Supplementary material for: Colchicine attenuates the electrical remodeling of post-operative atrial fibrillation through inhibited expression of immune-related hub genes and stabilization of microtubules
Source: Int J Biol Sci. 2023 Jun 4;19(9):2934–56. doi: 10.7150/ijbs.81961 (PMC10266076; doi:10.7150/ijbs.81961)
Supplement: Supplementary file 1 — Supplementary figures and tables. [file ijbsv19p2934s1.pdf]

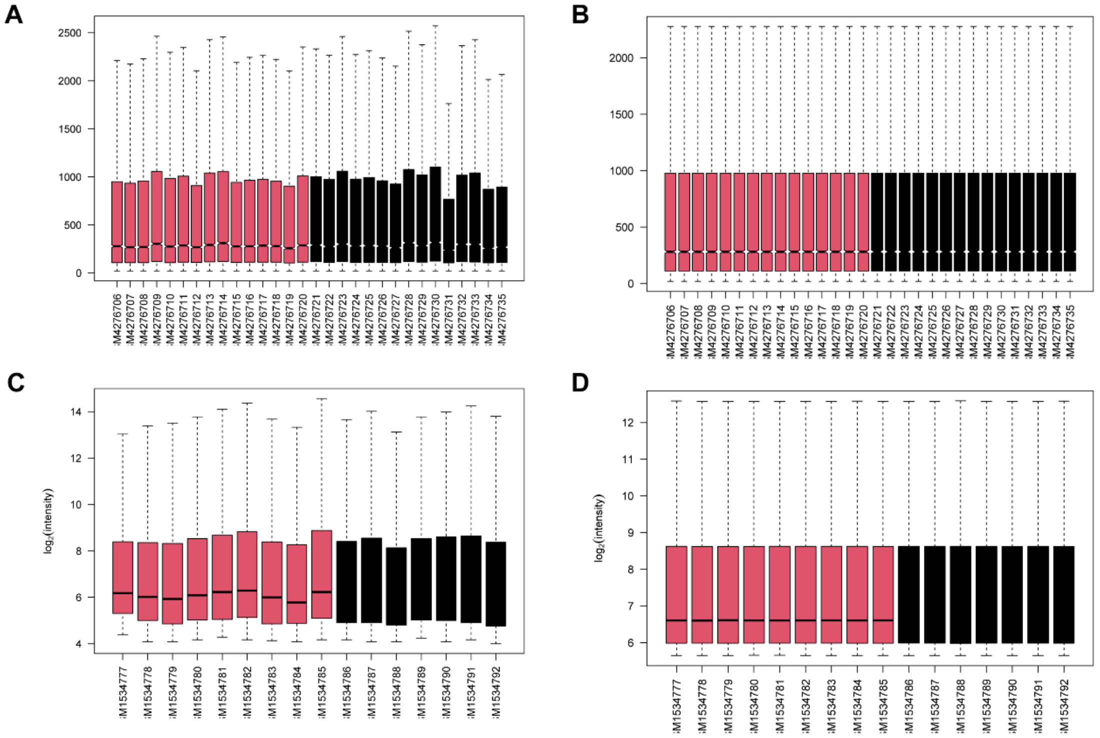

Supplemental Figure 1. Quality control of arrays. A. Box plot of the raw expression data of GSE143924. **B.** Box plot of the expression data of GSE143924 after RMA normalization. **C.** Box plot of the raw expression data of GSE62871. **D.** Box plot of the expression data of GSE62871 after backgroundCorrect, normalizeWithinArrays and normalizeBetweenArrays.

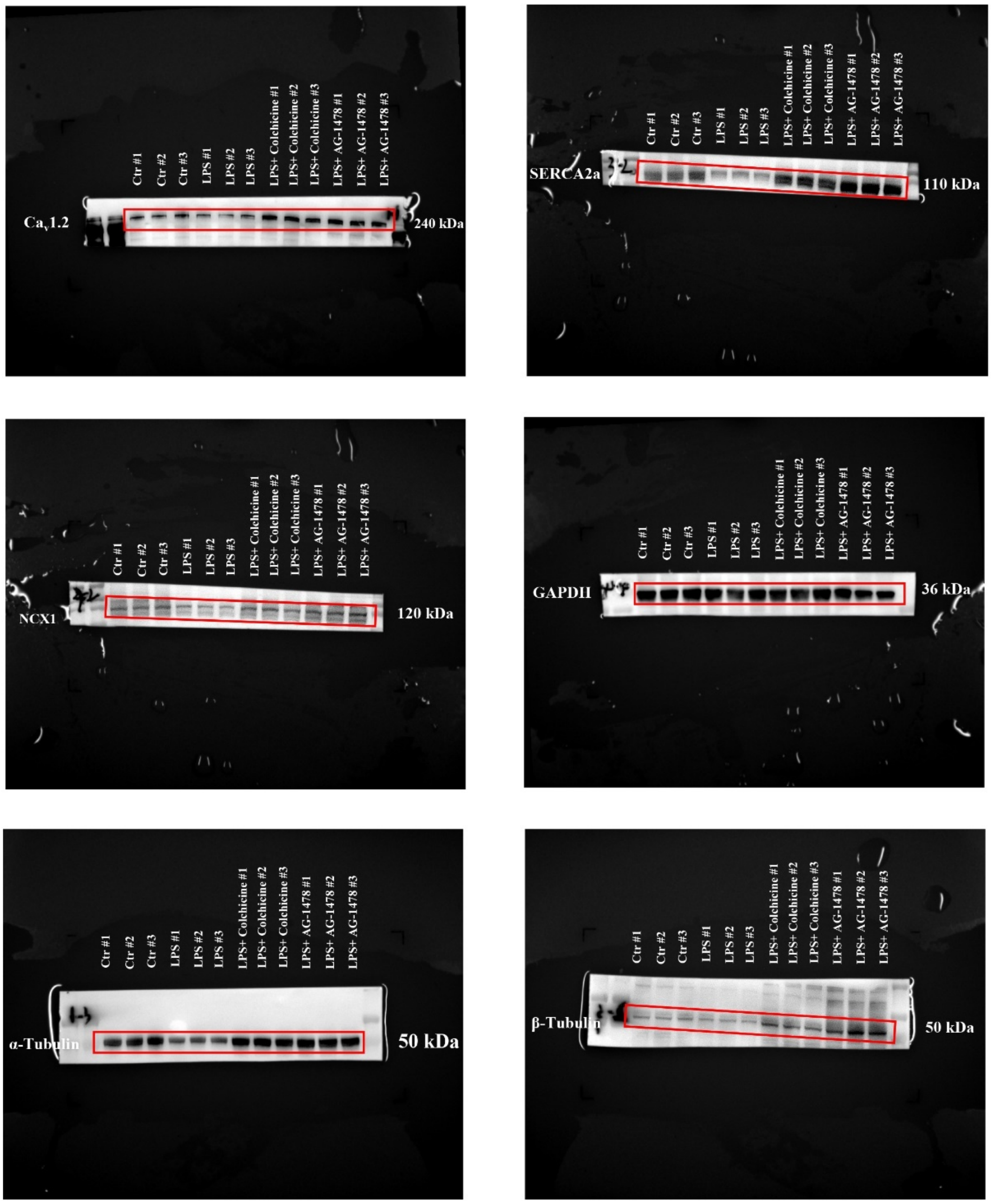

Supplemental Figure 2. Full length blots of Ca$_V$1.2, SERCA2a, NCX1, α-Tubulin, β-Tubulin and GAPDH expression in control, LPS, LPS + Colchicine, and LPS + AG-1478 groups. Red boxes indicate the cropped shown in Figure 7G.

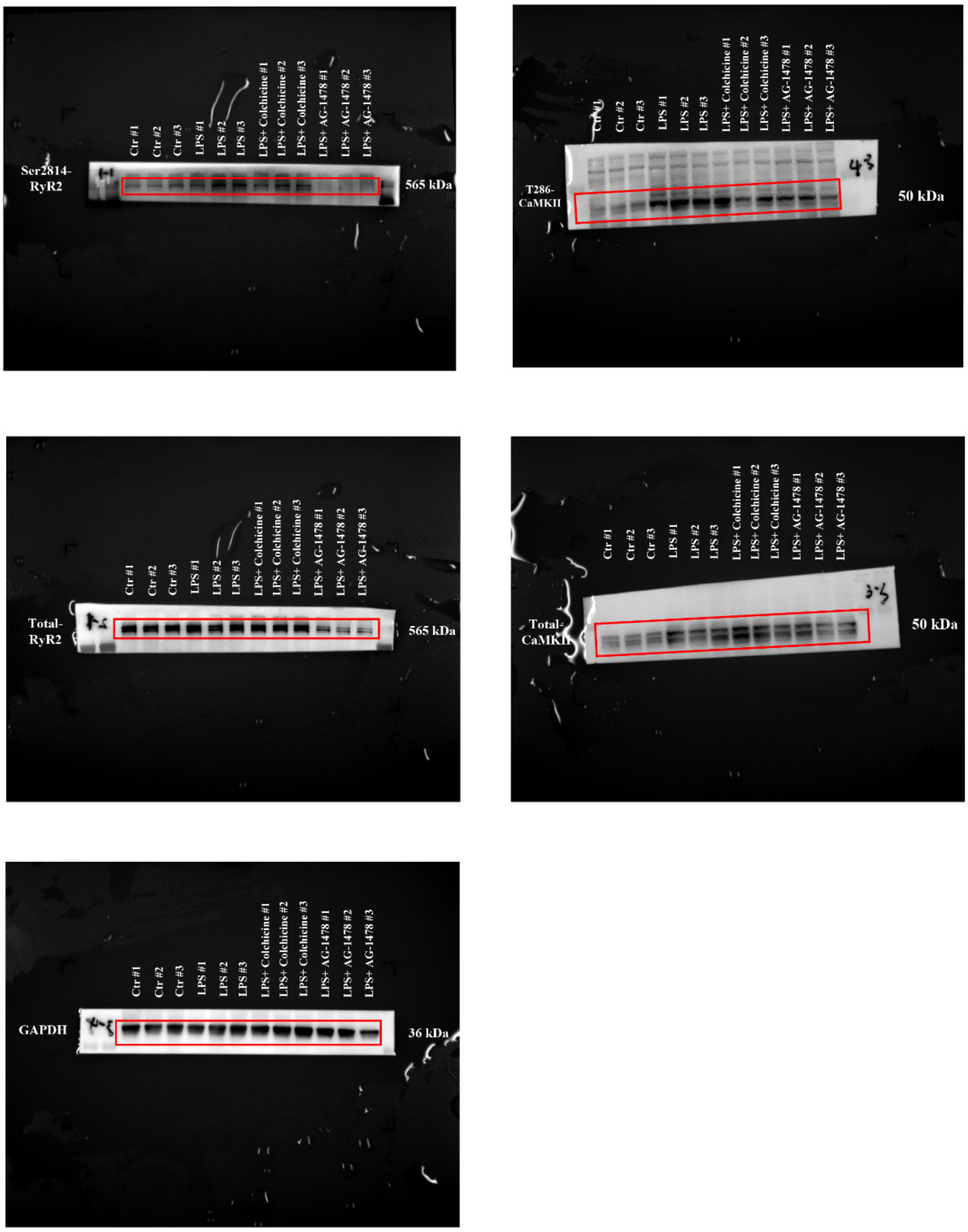

Supplemental Figure 3. Full length blots of Ser2814-RyR2, Total-RyR2, T286-CaMKII, Total-CaMKII and GAPDH expression in control, LPS, LPS + Colchicine, and LPS + AG-1478 groups. Red boxes indicate the cropped shown in Figure 7M.

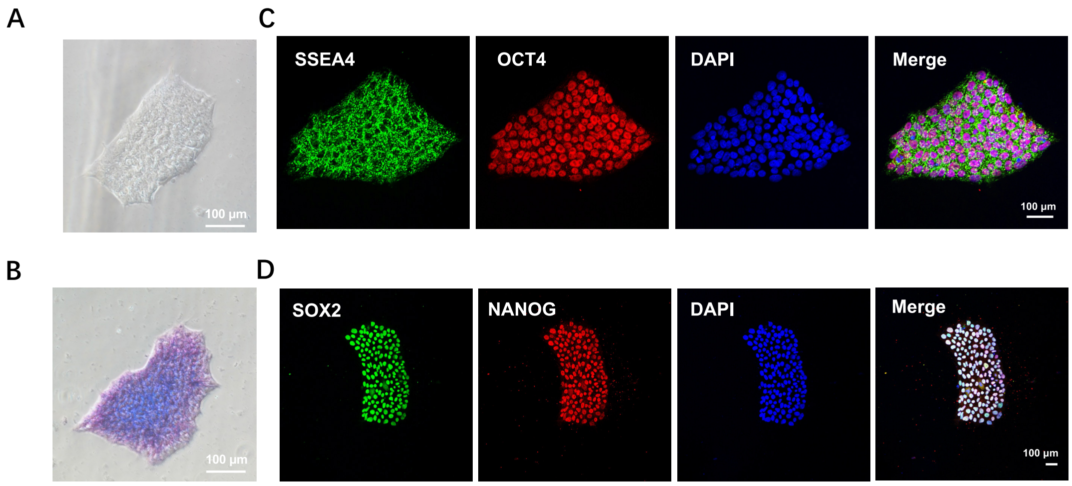

Supplemental Figure 4. Generation and characterization of iPSCs. A. Typical morphology of iPSCs derived from one healthy control subject. **B.** Alkaline phosphatase (ALP) staining of the iPSCs. **C-D.** Pluripotent staining of the iPSCs using SSEA4 (green), OCT4 (red), SOX2 (green) and NANOG (red). DAPI indicates nuclear staining (blue). Scale bar = 100 µm.

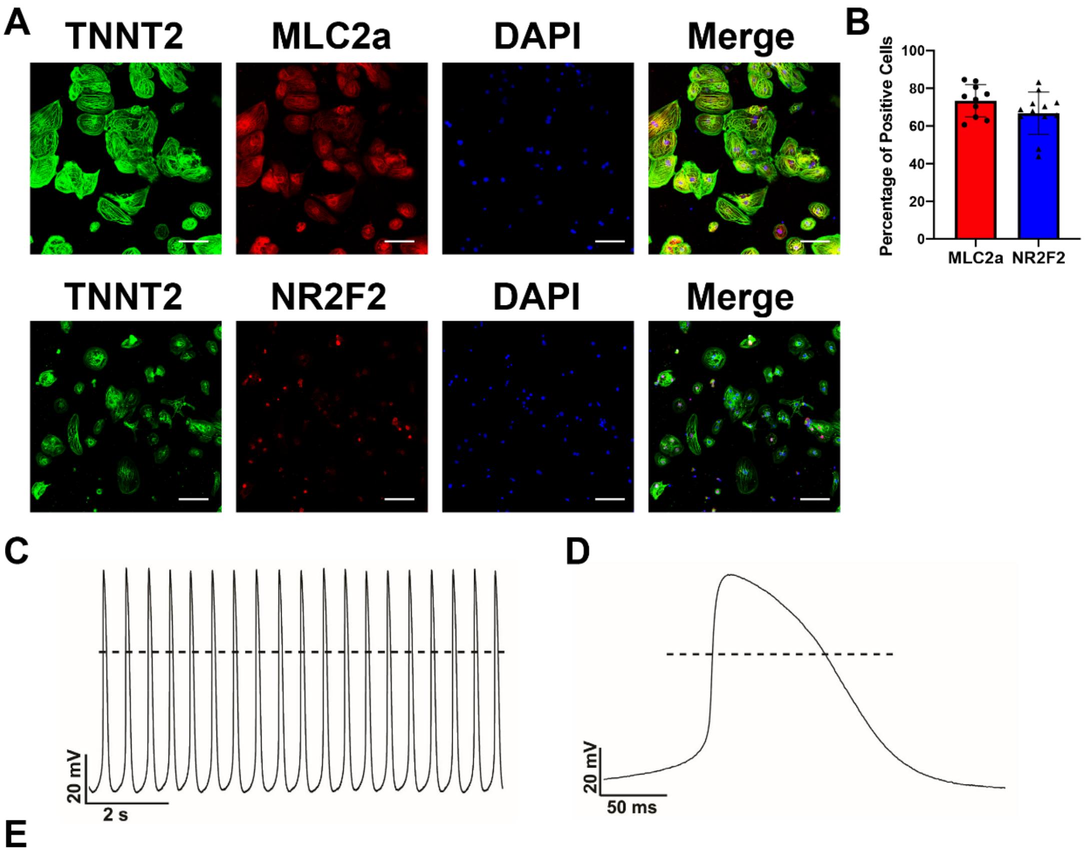

E

MDP (mV)	APA (mV)	APD_{90} (ms)	APD_{50} (ms)	V_{max} (V/s)	Beating rate (Beat per minute)	Repolarization fraction $(APD_{90}-APD_{50})/APD_{90}$
-56.7 ± 0.7	91.3 ± 1.3	153.9 ± 7.5	98.7 ± 5.3	10.7 ± 0.6	122.1 ± 8.2	0.36 ± 0.02

Supplemental Figure 5. Characterization of human iPSC-aCMs. A. Representative immunofluorescence staining of cardiac-specific marker TNNT2 and atrial-specific marker (MLC2a or NR2F2) in iPSC-aCMs. Scale bar, 100 µm. **B.** Bar graphs to show the percentage of MLC2a- positive and NR2F2-positive cells. 10 images were selected and quantified by ImageJ. **C-D.** Representative action potential tracings recorded by single-cell patch clamp from iPSC-aCMs. **E.** Summary table of key action potential parameters including maximal diastolic potential (MDP), action potential amplitude (APA), action potential duration 90 (APD_{90}), action potential duration 50 (APD_{50}), upstroke velocity (V_{max}), beating rate, repolarization fraction $(APD_{90}-APD_{50})/APD_{90}$ from iPSC-aCMs. The iPSC-aCMs exhibit unclear plateau phase, shortened APD, fast beating rate, and large repolarization fraction.

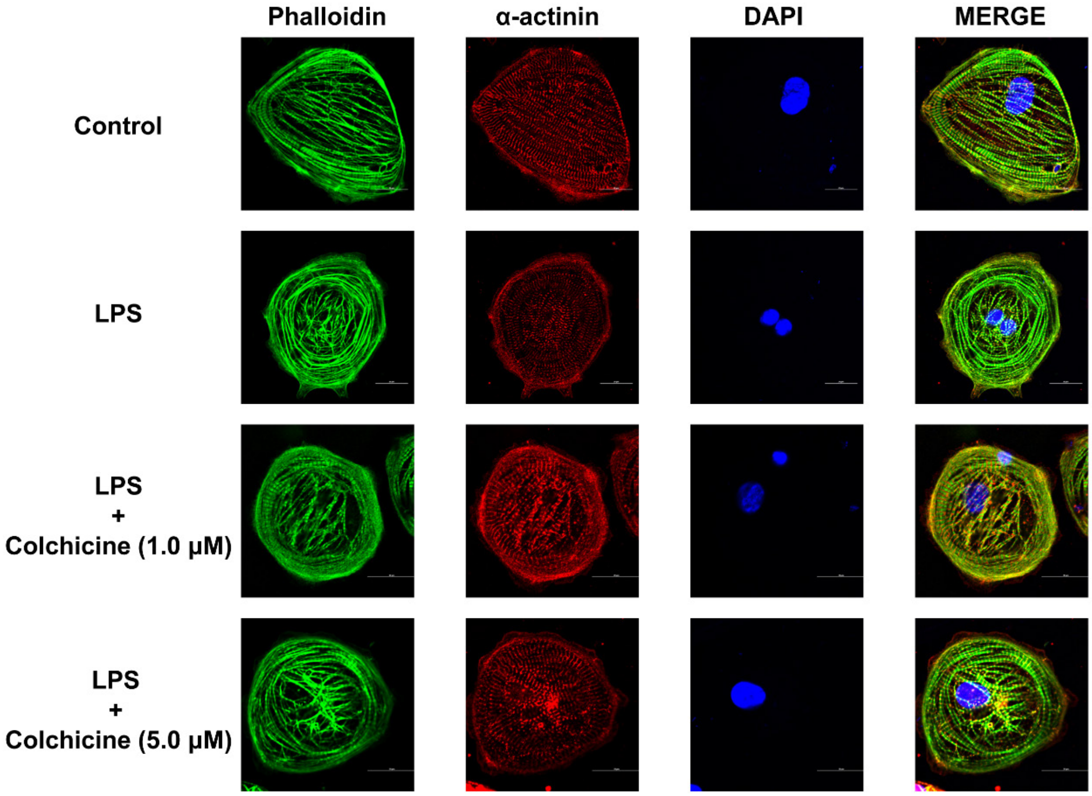

Supplemental Figure 6. Representative immunofluorescence staining of TNNT2 (green) and α-actinin (red) in baseline iPSC-aCMs (control), iPSC-aCMs treated with LPS alone (LPS group), iPSC-aCMs treated with LPS and low-dose colchicine (LPS + 1.0 μM colchicine group) and high-dose colchicine (LPS + 5.0 μM colchicine group). α-actinin is used as a marker for monomeric myofilament. DAPI indicates nuclear staining (blue). Scale bar, 100 μm.

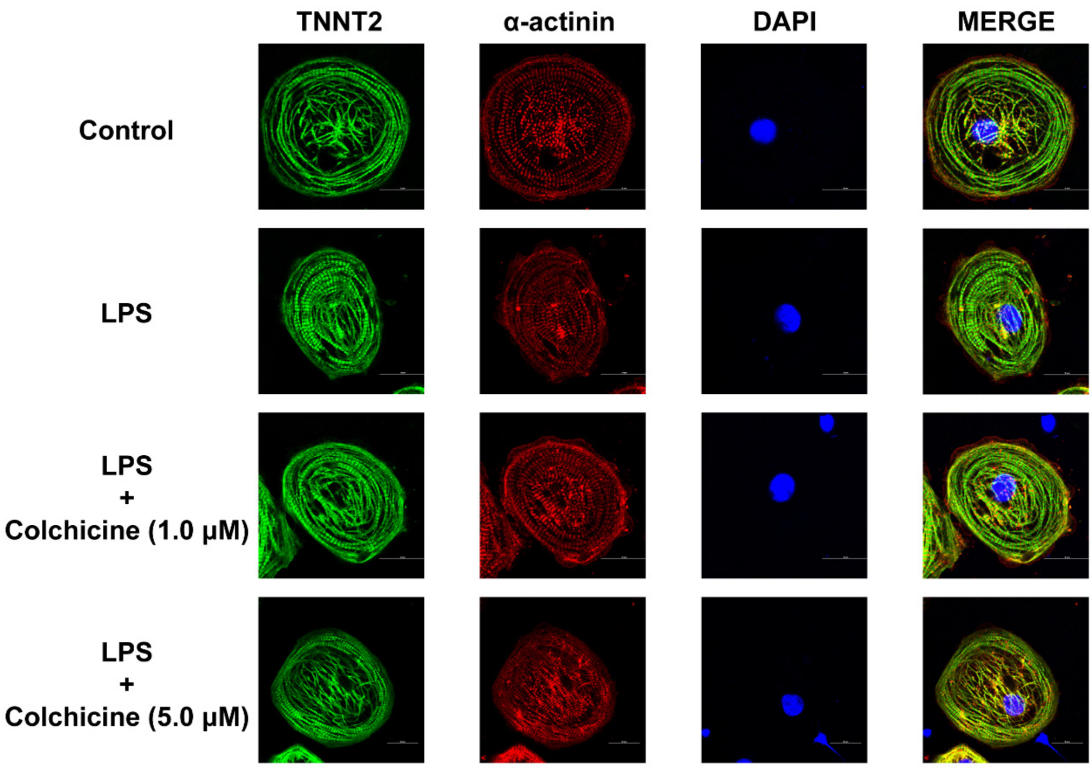

Supplemental Figure 7. Representative immunofluorescence staining of phalloidin (green) and α-actinin (red) in baseline iPSC-aCMs (control), iPSC-aCMs treated with LPS alone (LPS group), iPSC-aCMs treated with LPS and low-dose colchicine (LPS + 1.0 μM colchicine group) and high-dose colchicine (LPS + 5.0 μM colchicine group). Phalloidin and α-actinin are used as markers for poly-myofilament (F-actin) and monomeric myofilament, respectively. DAPI indicates nuclear staining (blue). Scale bar, 100 μm.

Supplemental Table 1. Detail information for GEO databases.

Accession	Platform	Type of RNA	Organism	Number of samples	Time	Country	Citation(s)
GSE143929	GPL25483	Expression profiling by array	Homo sapiens	Epicardial adipose tissue from 15 sinus patients and 15 atrial fibrillation patients undergoing operative surgery (15/15)	2020.06.03	France	Unpublished
GSE62871	GPL17077	Expression profiling by array	Homo sapiens	Right atrial appendage from 9 sinus patients and 7 atrial fibrillation patients undergoing operative surgery (9/7)	2014.10.30	France	PMID: 23644086

Supplemental Table 2. Primer sequences for qPCR in this study.

Gene	Forward (5'-3')	Reverse (5'-3')
VEGFA (mice)	CTGCTGTAACGATGAAGCCCTG	GCTGTAGGAAGCTCATCTCTCC
EGFR (mice)	AGAGCCAGAGCCAACCTCT	TGTGCCTTGGCAGACTTTCT
MMP9 (mice)	TCTGTCCAGACCAAGGGTACA	GCCTTGGGTCAGGCTTAGAG
CCL2 (mice)	TTAAAAACCTGGATCGGAACCAA	GCATTAGCTTCAGATTTACGGGT
CACNA1C (mice)	TCTCCAAATCCAAATTCAGC	ACTTCTGTGAGCCAGTGAGG
KCNJ2 (mice)	GATCTGACTGCTGAGGAGGA	GGTTGTTCCCTGTCTTGTTG
KCNH2 (mice)	GACCCTTTCCTGGCTTCACC	GACCAGCAGCAGGATGAGC
KCNQ1 (mice)	CTGAGAAAGATGCGGTGAAC	GGATATGGCGAAGACAGAGA
SLC8A1 (mice)	AGAAACCGAATGGAGAGACC	AGGGACCACGTAAACACAGA
SCN5A (mice)	TGTGATCGTCATGGCGTATGT	CAGGCCTGGAATAACTGAAATCG
KCND3 (mice)	CCTCCTCAAGCATCATAGCACT	TCTACCTGGATGCCTCTGTTC
GAPDH (mice)	AGGTCGGTGTGAACGGATTTG	TGTAGACCATGTAGTTGAGGTCA
VEGFA (human)	ACAACAAATGTGAATGCAGACCA	GAGGCTCCAGGGCATTAGAC
EGFR (human)	AACACCCTGGTCTGGAAGTACG	TCGTTGGACAGCCTTCAAGACC
MMP9 (human)	GCCACTACTGTGCCTTTGAGTC	CCCTCAGAGAATCGCCAGTACT
CCL2 (human)	TCAAACTGAAGCTCGCACTCT	GGGGCATTGATTGCATCTGG
CACNA1C (human)	AATCGCCTATGGACTCCTCTT	GCGCCTTCACATCAAATCCG
KCNJ2 (human)	GTGCGAACCAACCGCTACA	CCAGCGAATGTCCACACAC
KCNH2 (human)	CACCGCCCTGTACTTCATCT	AGGCCTTGCATACAGGTTCA
KCNQ1 (human)	GCGTCTCCATCTACAGCACG	GAAGTGGTAAACGAAGCATTTCC
SLC8A1 (human)	ACAACATGCGGCGATTAAGTC	GCTCTAGCAATTTTGTCCCCA
SCN5A (human)	TCACCGCCATTTACACCTTTG	GGTCCCGAAGGAAAGTGAACG
KCND3 (human)	GCATCCTCCCGGAGATCATC	CCGAGTCGTTGTCGTCCAT

GAPDH (human)	GGTCGGAGTCAACGGATTTG	CGGTGCCATGGAATTTGCC
